# Andean Mountain Building Did not Preclude Dispersal of Lowland Epiphytic Orchids in the Neotropics

**DOI:** 10.1038/s41598-017-04261-z

**Published:** 2017-07-07

**Authors:** Oscar Alejandro Pérez-Escobar, Marc Gottschling, Guillaume Chomicki, Fabien L. Condamine, Bente B. Klitgård, Emerson Pansarin, Günter Gerlach

**Affiliations:** 1 Department Biologie, Systematische Botanik und Mykologie, GeoBio-Center, Ludwig-Maximilians Universität, Menzinger Straße 67, D - 80638 Munich, Germany; 2 CNRS, UMR 5554 Institut de Sciences de l’Evolution (Université de Montpellier), Place Eugène Bataillon, 34095 Montpellier, France; 3 Identification and Naming department, Royal Botanic Gardens, Kew, TW9 3AB Surrey, UK; 4 Departamento de Biologia, Facultade de Filosofia, Ciências e Letras, Universidade de Sao Paulo, Ribeirao Preto, SP 14040-901 Brazil; 5 Botanischer Garten München, Menzinger Straße 61, D - 80638 Munich, Germany; 6 Identification and Naming department, Royal Botanic Gardens, Kew, TW9 3AB Surrey, UK

## Abstract

The Andean uplift is one of the major orographic events in the New World and has impacted considerably the diversification of numerous Neotropical lineages. Despite its importance for biogeography, the specific role of mountain ranges as a dispersal barrier between South and Central American lowland plant lineages is still poorly understood. The swan orchids (*Cycnoches*) comprise *ca* 34 epiphytic species distributed in lowland and pre-montane forests of Central and South America. Here, we study the historical biogeography of *Cycnoches* to better understand the impact of the Andean uplift on the diversification of Neotropical lowland plant lineages. Using novel molecular sequences (five nuclear and plastid regions) and twelve biogeographic models, we infer that the most recent common ancestor of *Cycnoches* originated in Amazonia *ca* 5 Mya. The first colonization of Central America occurred from a direct migration event from Amazonia, and multiple bidirectional trans-Andean migrations between Amazonia and Central America took place subsequently. Notably, these rare biological exchanges occurred well after major mountain building periods. The Andes have limited plant migration, yet it has seldom allowed episodic gene exchange of lowland epiphyte lineages such as orchids with great potential for effortless dispersal because of the very light, anemochorous seeds.

## Introduction

Neotropical landscape and biodiversity have long drawn the attention of naturalists^1, 2^. The tropical Andes are of particular interest as the world’s premier biodiversity hotspot, with both an extraordinary species richness and a remarkable level of endemism^3–5^. The combination of molecular phylogenies with species distributions and the fossil record has uncovered different biotic and abiotic factors that fostered diversification in the Neotropics^6–10^. Biogeographical studies applying modern phylogenetic methods on Neotropical plant clades (e.g. *Begonia* ^11^, *Cyathostegia* ^12^, *Dioscorea* ^13^, *Heliotropium* ^14^, *Lupinus* ^15, 16^, Arecaceae^17^, Orchidaceae^18^, Rubiaceae^6^) have generally demonstrated the importance of geological processes such as mountain building and establishing the Isthmus of Panama for the diversification of Neotropical plants^19^.

One of the most biologically important abiotic processes in the diverse geological history of the Americas is the rise of the Andes^7, 14, 20^. Andean mountain building was driven by plate tectonic re-adjustments that started during the Palaeogene and continued until the Pliocene^7^. The fossil record (e.g. palynological^3, 21^ and geological data: isotope measurements^22^, sediment loads, apatite fission-track data^7^) collectively indicate that the Andean uplift was a partially constant process punctuated by periods of intensified mountain building. Two of the most intense uplift periods occurred around 12 (mid-Miocene) and 4.5 million years ago (Mya; early Pliocene^7^). During these periods, the Northern Andes reached elevations as high as 4500 m in the Pliocene, whereas the Central Andes already peaked an altitude of 4500 m during the mid-Miocene^7, 23^.

Newly formed mountain ranges may had a strong impact on the adjacent Amazonian landscapes and the inhabiting organisms due to the transformation of its drainage systems^24^. The Andes also influenced local and regional climates by forming the only barrier to atmospheric circulation in the Neotropics^25, 26^. The rise of the Andes led to the formation of island-like habitats and of local microclimates and soil conditions that eventually fostered species diversification^15, 27, 28^. At the same time, the Andes provided physical and/or ecological barriers to species dispersal and migration. For instance, the efficiency of the Northern Andes as migration barrier is shown for Andean centred woody species of *Crematosperma*, *Klarobelia*, *Malmea*, and *Mosannona* (Annonaceae^29^). Propagules are dispersed by animals in these lineages^30^, and none of their constituent species occur in both east and west of the Andes mountain range^29^.

Recent phylogenetic studies provide solid evidence for the important role of Andean uplift in diversification of highland-dwelling plant groups (e.g. *Lupinus* ^15^; *Bartsia* ^31^; centropogonid Campanulaceae^9^). However, the impact of such orogenic processes for the lowland flora is still poorly understood^4, 6, 32^. Thus, the question remains whether Andean uplift has indeed been an abiotic barrier to migration for epiphytic lineages such as lowland orchids and bromeliads, both being important components of Neotropical forests.

Epiphytic diversity is dramatically greater in the Neotropics than in any other tropical region around the world^33, 34^, being twice as high than, for instance, in Australasia^35–38^. Several traits shared by Neotropical epiphytic taxa, and related to their reproductive biology, may explain such overwhelming differences in diversity. One of the most prominent shared traits is the lightness and the very small size of the propagules, occasionally with highly elaborated surfaces (e.g. bromeliads, ferns, orchids, *Pinguicula* ^35^). The potential of dust-like seeds for rare longer distance dispersals by wind might be greater compared with other plants with propagules locally dispersed by animals (e.g. Araceae^39^). Indeed, detailed studies on epiphyte orchid seed dispersal and gene flow provide evidence for the rarely long distance exchange of orchid propagules between geographical areas^40–42^. Nonetheless, the frequency and tempo of lowland epiphyte migrations across geological barriers such as the Andes remains largely unknown. Well-sampled phylogenies for epiphyte clades have been lacking to address this issue.

Several wind-dispersed plant lineages (e.g. *Begonia* ^11^; bromeliads^43^) span across the Neotropical region, many of which are restricted to lowland elevations. One example is the orchid tribe Cymbidieae comprising *ca* 3900 species that are mostly distributed in the Neotropics (but with few representatives in the Old World Tropics^44^). To Neotropical Cymbidieae belong the swan orchids (*Cycnoches*) that are known for the striking sexual dimorphisms^45^ and pollination syndrome^46^. Molecular phylogenetics and morphological studies conducted to date confirm the inclusion of *Cycnoches* within Catasetinae^47–49^ and its sister group relationship to *Mormodes* ^45, 50, 51^.

*Cycnoches* encompasses 34 species of epiphytes^52^ that are distributed from Southern Mexico to Central Brazil and Bolivia. Its species are best represented in the Amazonian forests of Brazil, Ecuador, and Peru, but also in the Llanos and Caribbean regions of Colombia and Venezuela^52, 53^. They commonly inhabit lowland tropical wet forests, ranging from 0 to 800 m. *Cycnoches* species are pollinated by male euglossine bees, which collect volatile compounds produced by flowers but also from other sources (e.g. rotten wood, faeces^46^). They have rather a restricted geographical range, and most of them are distributed in single biogeographical areas. Nevertheless, one species (*C*. *chlorochilon*) is distributed on both sides of the Northern Andes^52^.

Because of the striking sexual system they have evolved^45^, swan orchids have long attracted the attention of several prominent naturalists including Charles Darwin^54^. Despite this long interest, previous phylogenetic studies have only been included up to three species of *Cycnoches* probably because of their scarceness both in the wild and in herbarium collections^47, 48, 50, 51, 55^. The lack of a solid and well-sampled phylogeny of *Cycnoches*, has precluded addressing specific questions concerning the role of Andean uplift in their biogeographical history, a question urged by their distribution on the two sides of the Andes. The narrow geographical distribution of almost all extant *Cycnoches* species and their restricted habitat preference given (i.e. lowland wet forests), we expect the swan orchids diversification to be strongly influenced by the Andean uplift. In particular, we hypothesize that Andean uplift restricted genetic exchange in *Cycnoches*, as already reported for other plant lineages^6, 13, 29^. To test this hypothesis, we generated a strongly supported 5-loci phylogeny, sampling 23 out of 34 accepted *Cycnoches* species and comprising its known diversity and distribution, and performed models of biogeographical analyses.

## Results

### Phylogeny of Cycnoches

Our phylogeny comprised 23 out of 34 accepted species of *Cycnoches*. Table S5 provides detailed alignment descriptions. The concatenated nuclear alignment was 2395 bp in length and included 310 parsimony informative sites, and the concatenated plastid alignment was 2419 bp and comprised 171 parsimony informative positions. Independently derived nuclear and plastid phylogenies revealed topologies with conflicting and highly supported phylogenetic placements. PACo analysis revealed 22 potential outlier OTUs (see below; Figs S1 and S2) belonging to eleven species of *Cycnoches* (19 outliers), one species of *Dressleria*, and two of *Mormodes*. After inspection of the potentially conflicting OTUs placement in nuclear and plastid phylogenies (Fig. S2), 20 outliers were confirmed as conflicting terminals (Tab. S6) and were excluded from the concatenated nuclear-plastid DNA matrix. Thus, only two terminals (i.e. *Dressleria severiniana* and one representative of *C*. *lehmannii*) were misclassified by PACo as conflicting (Figs S1 and S2; see Appendix S1 for a detailed explanation on outlier handling).

**Figure 1:**
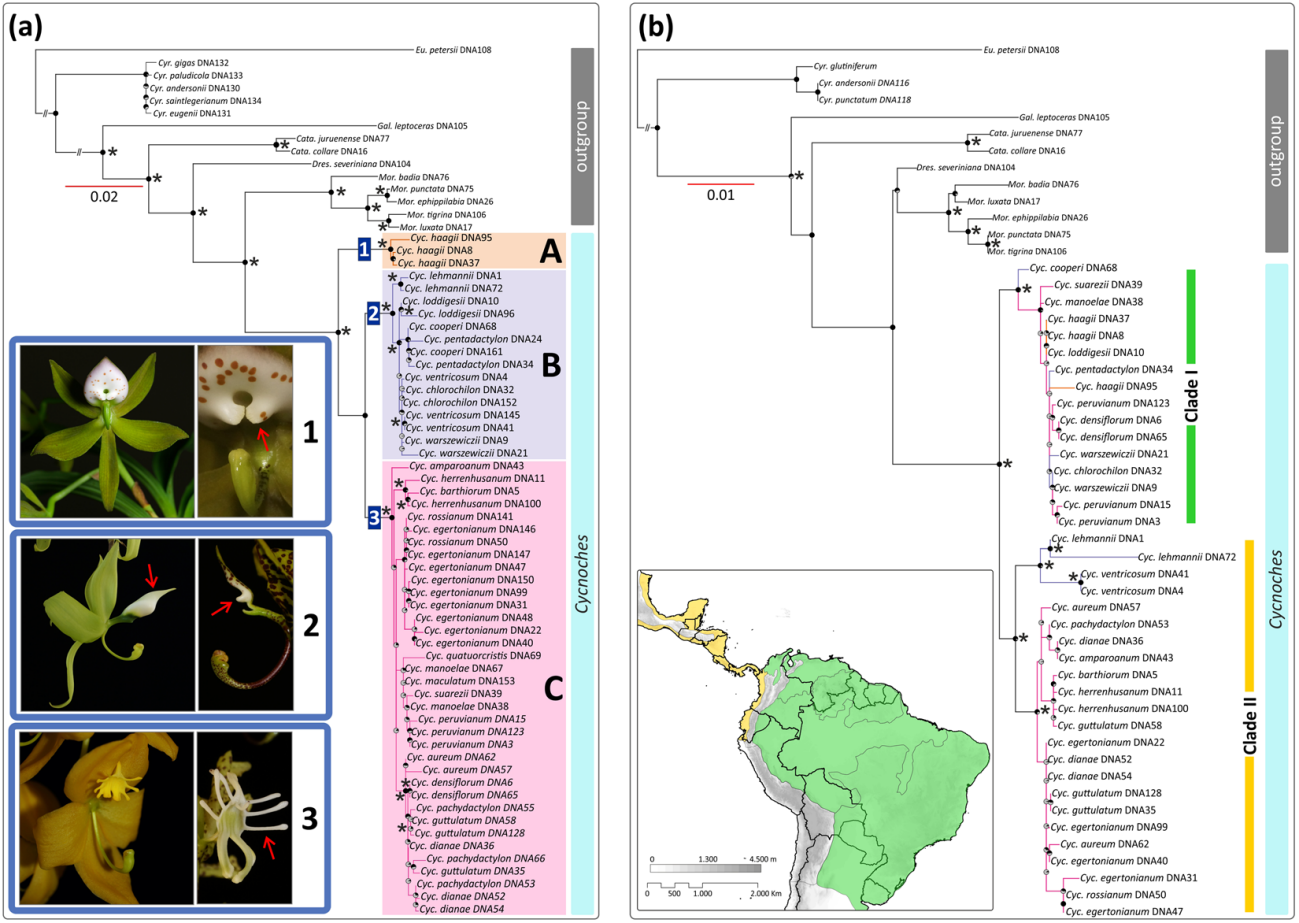
Best scoring ML tree of *Cycnoches* obtained from concatenated (**A**) nuclear ETS, ITS, *Xdh* and (**B**) plastid *trn*S–*trn*G, *ycf*1. Node charts indicate Bootstrap Support (MLBS > 75), in where fully red diagrams indicate MLBS of 100. Numbers at nodes indicate Bayesian Posterior Probability (PP > 0.95). Representatives of each clade are shown in pictures: (1) *C*. *haagii*; (2) *C*. *ventricosum* (left) and *C*. *pentadactylon* (right); (3) *C*. *herrenhusanum* (left) and *C*. *peruvianum* (right). The red arrows indicate apomorphies of every clade: in (1) fleshy oblong, curved pair set of calli located towards the base of the labellum, (2) labellum blade entire to 4-lobed, and (3) labellum with 8–10 marginal projections. Geopolitical boundaries map generated by ArcMAP software (http://www.esri.com), using layers freely available at http://www.diva-gis.org/Data. Photos: O. Pérez & G. Gerlach.

**Figure 2:**
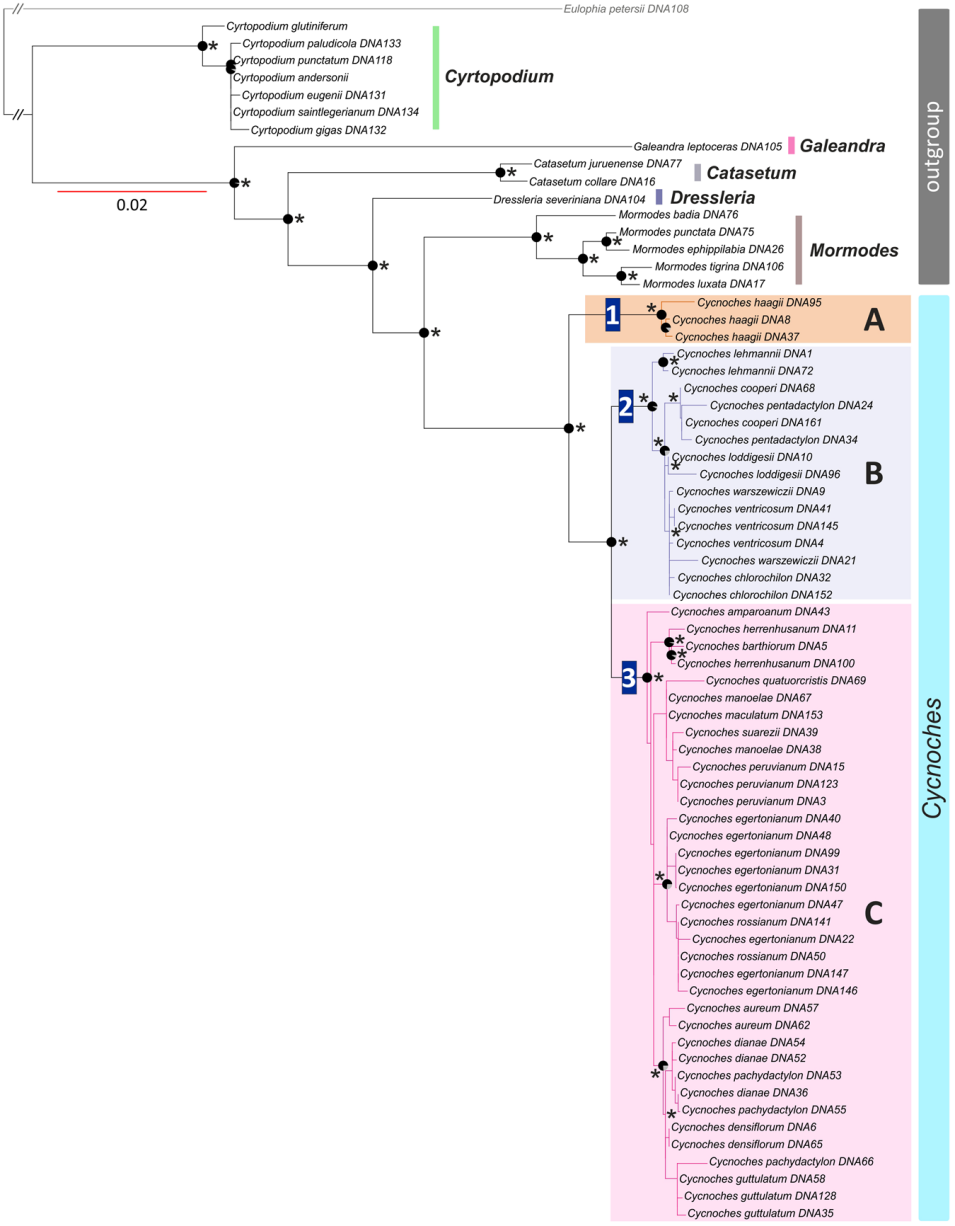
Best scoring ML tree of *Cycnoches* obtained from concatenated, non-conflicting nuclear ETS, ITS, *Xdh* and plastid *trn*S–*trn*G, *ycf*1. Node charts indicate Bootstrap Support (MLBS > 75), in where fully red diagrams indicate MLBS of 100. Numbers at nodes indicate Bayesian Posterior Probability (PP > 0.95).

Within *Cycnoches*, the nuclear phylogeny recovered three maximally supported clades (A, B, C; Fig. 1). Clade A included all sequenced accessions of *Cycnoches hagii* and was recovered as sister group of the remaining species of *Cycnoches* comprising clades B and C. By contrast, the plastid phylogeny showed two main maximally supported clades (namely I and II), each including a set of intermingled species from clades A, B, and C. The best ML tree inferred from the non-conflicting, concatenated nuclear and plastid dataset, showing the internal phylogenetic relationships of *Cycnoches*, is presented in Figure 2. Almost all internal nodes of the backbone phylogeny were highly, if not maximally supported by Maximum Likelihood Bootstrap Support (MLBS) and Posterior Probability (PP) values. *Cycnoches* segregated into three main lineages (clades A, B and C). Clade A (i.e. all specimens of *C*. *haagii*) was sister group of the remaining species of *Cycnoches* clustering in clades B and C.

### Molecular dating of Cycnoches

**Figure 3:**
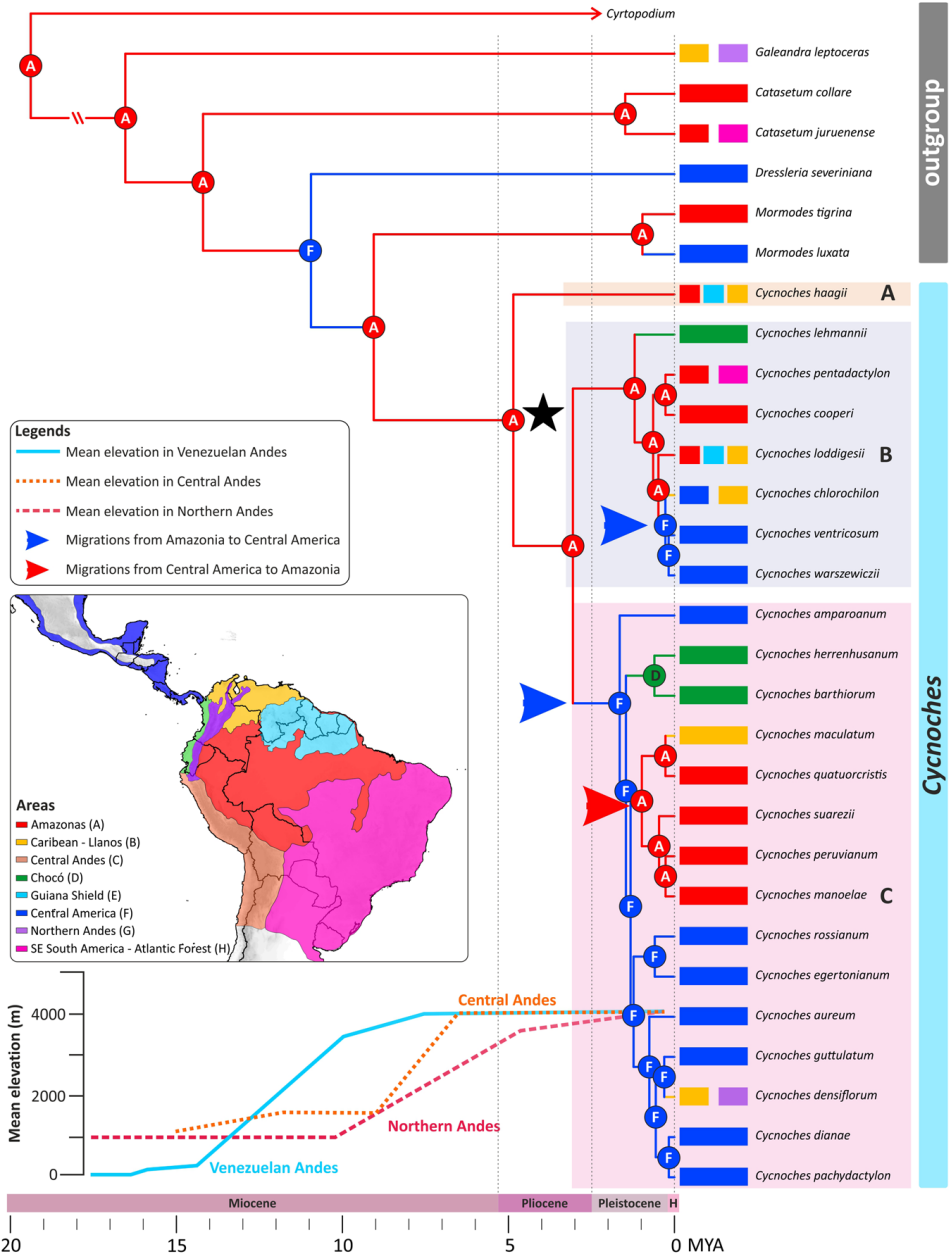
Chronogram for *Cycnoches* obtained under a relaxed clock model, applied to a non-conflicting, concatenated nuclear (ITS, ETS, *Xdh*) and plastid loci (*trn*S–*trn*G, *ycf*1). Age estimates, including maximum and minimum intervals for all nodes, are provided in Figure S4. Time scale is provided in million years ago (Mya). Node charts correspond to ancestral areas estimated under the BayArea-Like* model, including founder event process (*J*). Geographical distribution in coded biogeographical areas of sampled species is shown in front of tree terminals. Pink, orange, and blue lines indicate mean elevations (m) on Colombian, Central and Venezuelan Andes, respectively (adapted^7^). (Inset) Coded areas used for biogeographical analysis. Geopolitical boundaries map generated by ArcMAP software (http://www.esri.com). Political divisions and elevation data from DIVA-GIS (http://www.diva-gis.org/gdata).

Comparison of marginal likelihood estimates (MLE) of tree priors and clock models revealed that an uncorrelated molecular clock combined to a birth-death tree prior with incomplete sampling best fitted our data (MLE of –14244.47; Tab. 1). Nevertheless, Bayes factors (BF) clearly rejected the strict clock models (BF = 72, 45, and 40 for all tree priors), but did not provide strong evidence to support an incomplete sampling birth-death model *vs* a standard birth-death model (BF = 1.08; Tab. 1). Analysis of the log file produced by dating analyses under the relaxed clock and tree models are also shown in Table 1. Overall, they yielded CV values between 0.35 and 0.42 (indicating there was among branch rate heterogeneity, which argued for the use of a relaxed molecular clock). Therefore, results obtained from a dated phylogeny with relaxed molecular clock and birth-death standard speciation model (Tab. 1) are presented only (Figs 3 and S4).

**Table 1:** Results of the molecular dating analyses comparing tree speciation models for relaxed and strict molecular clocks.

	Tree prior	MLE (SS)	CV	Yule	BD	BD sampling	Yule	BD	BD sampling
Strict clock	Yule	−14280.93	—	—					
	BD	−14267.36	—	27.14	—				
	BD sampling	−14264.73	—	32.4	5.26	—			
Relaxed clock	Yule	−14259.53	0.42	42.8	15.66	10.4	—		
	BD	−14245.01	0.35	71.84	44.7	39.44	29.04	—	
	BD sampling	−14244.47	0.35	72.92	45.78	40.52	30.12	1.08	—

Marginal likelihoods of estimates were performed under BEAST and the stepping-stone sampling (SS). BD stands for birth-death model.

A chronogram showing absolute ages estimated under a relaxed clock is presented in Figure 3 (see also Fig. S4 for the 95% confidence intervals) and shows that *Cycnoches* and *Mormodes* shared a common ancestor during the beginning of the late Miocene (9.1 Mya ± 3). Diversification of *Cycnoches* took place around 5 Mya ± 2 at end of the late Miocene. The split between *Cycnoches* clades B and C occurred during late Pliocene (3 Mya ± 2), whereas both clades were estimated to be of Pleistocene ages (1.2 and 1.6 Mya ± 1, respectively).

### Ancestral area estimations

Table 2 provides ML statistics for the biogeographical models as inferred in BioGeoBEARS. The best fitting model was the BAYAREA star, including the founder-event speciation. This model revealed Amazonia as the most likely ancestral area of *Cycnoches* (Figs 3 and S5). The MRCA of clades B and C was reconstructed to have inhabited in the Amazonian region. The MRCA of clade C occurred in Central America, whereas the MRCA of clade B inhabited in Amazonia region.

**Table 2:** Results of the biogeographic analyses performed with DEC, DIVA-like and BayArea-like models as implemented in BioGeoBEARS.

Model	LnL	AIC	AICc	AIC weighted
DEC	−119,3054256	242,6108513	242,9744877	1,06E-08
DEC + J	−110,4860209	226,9720418	227,7220418	2,63E-05
DEC*	−106,2412542	216,4825085	216,8461448	0,004984487
DEC + J*	−103,9600773	213,9201546	214,6701546	0,017948515
DIVA	−125,2827663	254,5655327	254,929169	2,68E-11
DIVA + J	−123,1895028	252,3790055	253,1290055	7,99E-11
DIVA*	−104,1616852	212,3233704	212,6870068	0,039880977
DIVA + J*	−104,9727468	215,9454937	216,6954937	0,006519762
BayArea	−131,2239925	266,4479849	266,8116213	7,04E-14
BayArea + J	−113,2503658	232,5007317	233,2507317	1,66E-06
BayArea*	−119,7393191	243,4786382	243,8422745	6,85E-09
BayArea + J*	**−100,011714**	**206,0234279**	**206,7734279**	**0,930638293**

We inferred three independent trans-Andean migration events between Amazonia and Central America. The first migration from Amazonia to Central America took place towards the late Pliocene (±1 Mya), after the divergence of MRCAs of clades B and C. A second migration from Central America to Amazonia took place around mid-Pleistocene (±1 Mya) by the MRCA of *C*. *maculatum*, *C*. *manoelae*, *C*. *peruvianum*, *C*. *quatuorcristis*, and *C*. *suarezii*. The last biotical exchange from Amazonia to Central America was dated to late Pleistocene (±0.5 Mya) with the MRCA of *C*. *chlorochilon*, *C*. *ventricosum*, and *C*. *warszewiczii*.

## Discussion

### Influence of Andean orogeny on the biogeography of a Neotropical epiphyte group

Our study provides a solid phylogenetic framework for the evolution of *Cycnoches* in time and space. Central America has been considered the most likely region of *Cycnoches* origin, probably because of its locally elevated species richness as compared to other areas in the Neotropics^56^. However, our analyses reject this evolutionary scenario, which instead support a South American origin of *Cycnoches* in the late Miocene (*ca* 5 Mya ±2; Figs 3 and S5). The estimated ancestral area of *Cycnoches* MRCA largely reflects the current distribution of early diverging lineages such as *C*. *haagii*, a species today distributed in Amazonia and the Guiana Shield.

The early diversification of *Cycnoches* has taken place well after the most intense mountain building events of the Northern and Central Andes *ca* 12 and 10 Mya, respectively^7, 57–59^, and our results indicate a less limiting role of the Andes as a biogeographic barrier for the diversification of *Cycnoches*. Two migrations from Amazonia to Central America and one reverse colonisation event imply rare, ancient dispersals across the Andes during last 5 million years (Fig. 3). During the early Pliocene (i.e. the period when *Cycnoches* has started to diversify), the Northern Andes of Colombia and Venezuela have already reached elevations up to at least 3000 m^7^. Moreover, three migrations from Amazonia to Central America and back, respectively, took place ~2 Mya, when the Northern and Central Andes already peaked at a mean elevation of 4000 m (see mean Andean elevation displayed in the inset of Fig. 3). Such dispersals might have been assisted by vegetation expansions and contractions due to dramatic temperature changes after the mid-Miocene climatic optimum^6, 27^ that are known to have affected the distribution ranges of lineages in Central and South America^60^. These changes in vegetation ranges might have shortened the distances between populations distributed in either side of the Andes.

Similar trans-Andean migrations are reported for bromeliads^43^, ferns^32^, and more recently in Cymbidieae orchids^18^. In Bromeliaceae (subfamily Bromelioideae), direct migrations from the Brazilian Shield towards Central America have taken place around 7 Mya^43^, a period where Northern Andes reached a paleoelevation of ~2000 m^7^. Furthermore, biotical exchanges between South and Central America have also been reported in the fern complex *Jamesonia-Eriosorus* ^32^. Here, migrants from the Brazilian coast colonized and further established in Central Andes, and from there subsequently migrated towards Central America during the late Pleistocene^61^. In Cymbidieae orchids, direct migrations from Amazonia to Central America in Catasetinae and Zygopetalinae were inferred to have taken place early during the evolutionary history of such lineages during middle Miocene (~15 Mya^18^). During this time period, Colombian and Venezuelan Andes peaked a mean elevation of *ca* 1100 m^7^.

The Andes does not appear to be a limiting barrier of genetic exchange for Neotropical epiphyte lineages such as *Cycnoches* compared with zoochorous groups (e.g. Annonaceae^29^, Rubiaceae^6^). The rapid colonisation of Central America and Amazonia may be related to the biology of the group having anemochorous seed dispersal and an epiphytic habit. Most orchid seeds are characterized by their minute size and reduced weight, as well as by their elaborated seed coats^42, 62^. These traits allow them to easily remain airborne for extended periods of time and might promote rare long distance dispersals over geographical barriers^62^. Our results of ancestral area estimations provide support for these seldom migrations across the Andes. Moreover, *Cycnoches* species might have good dispersal potential as well because of their seeds, which are between 100–300 µm long, 50–60 µm wide, and about 3–4 µg weight^42, 63^.

### Phylogenetic conflict between nuclear and plastid phylogenies in Cycnoches

Our study brings important insights for the species relationships within *Cycnoches*. Previous phylogenetic studies about Catasetinae have included not more than three species of *Cycnoches* ^47, 48, 50, 51, 55^, hence keeping internal relationships (and corresponding conflicts, see below) of the lineages unresolved. Serious phylogenetic incongruence between nuclear and plastid tree topologies of Catasetinae has been firstly identified by Pérez-Escobar and colleagues^64^, but the plausibility of the trees were not discussed before.

All major clades in our nuclear phylogeny are consistent with morphological concepts of *Cycnoches* (Fig. 1a). *Cycnoches haagii* (Clade A) differs from other *Cycnoches* species by the fleshy oblong, curved pair set of calli located towards the base of the labellum. Clade B includes species with conspicuously large male flowers and an entire to 4-lobed labellum blade. Clade C comprises species with proportionately small male flowers and labella with 8–10 marginal projections^53^. The consistency between molecules and morphology is challenged by an apparent correlation between the plastid molecular tree and the distributions of particular lineages (Fig. 1b): Clade I comprises species occurring mostly in Amazonia, Caribbean-Llanos, and Guiana shield regions (except *C*. *chlorochilon* and *C*. *warszewiczii* present in Costa Rica and Panama). Clade II, in turn, includes species exclusively distributed in Central America and Chocó regions.

Topological incongruence between phylogenies derived from different genomes is a widespread phenomenon in phylogenetic inferences^65–69^. Examples from several angiosperm lineages (e.g. Araceae^39^, Asteraceae^70^, Saxifragaceae^71^) have long revealed conflicting patterns, in which the nuclear phylogeny is in accordance with morphology, whereas the plastid relationships correlate to geographical distributions. The nuclear-morphological and plastid-geographical phylogenetic relationship patterns recovered in *Cycnoches* follow this phenomenon, which might be associated to the genetic exchange promoted by seeds *vs* pollen. More importantly, the differential levels of gene flow facilitated by seed and pollen dispersal, and the transmission modes of plastids, may explain the incongruences observed between nuclear and plastid datasets^67^. Here, the lower genetic exchange promoted by seed dispersal^72, 73^ support the conserved geographical structure in the (maternally inherited^74, 75^) plastid phylogeny, while the congruence of morphological traits with the (biparentally inherited) nuclear tree might be related with the higher levels of genetic exchange facilitated by pollen^67, 76^.

Another process with explanatory power for the nuclear-plastid conflict observed in the clade is hybridization. Euglossine-bee-pollinated orchids such as *Cycnoches* produce a blend of volatile compounds, which attracts male Euglossine bees, and pollination takes place while bees collect such compounds produced by specialized tissues in the flower^77^. Species-specific production of floral blends and therefore attraction of a unique set of pollinator(s) has been accounted as an isolative reproductive barrier in Euglossine bee pollinated orchids^46, 78^. Nevertheless, intra-specific variation of fragrances produced by the flower has been reported in several orchid lineages such as *Stanhopea* ^79^ and even *Cycnoches* ^80^. Fragrance variation may result on attraction of a set of pollinators that are shared by species co-occurring in the same biogeographical region (e.g. *C*. *dianae*, *C*. *guttulatum*, and *C*. *pachydactylon* ^80^) with similar composition of the fragrance profile, providing an opportunity to hybridization to occur. Little is known about pollinators of *Cycnoches*, but our own observations of pollinator sharing between species and presence of polymorphic species may argue for the possibility of past hybridization processes.

## Conclusion

Based on a solid, comprehensively sampled phylogeny we provide macroevolutionary evidence for a South American origin of *Cycnoches*. Our biogeographical analysis indicates colonization of Central America via a direct migration from the Amazonian basin. More importantly, the analyses support three recent trans-Andean, bidirectional migration processes between Central America and Amazonia, which is indicative for plausibility of rare long distance dispersals in lowland epiphytic orchids, and suggest a less limiting role of the Andean barrier on swan orchids migration. Consequently, our study enlightens the semi-permeability of Andean mountain building on the range evolution and diversification of lowland Neotropical epiphytic lineages. Our study paves the way for detailed studies on the effect of Andean orogeny in population gene flow of epiphytic orchids occurring at both sides of the range.

## Material and Methods

### Taxon sampling, DNA sequencing, and phylogenetic analyses

Species names, geographical origins, voucher specimens, and GenBank accession numbers of sequences included in phylogenetic analyses are provided in Table S1. Our study builds-up upon previous DNA data matrices^43, 51, 63^. Genomic DNA was extracted from herbarium and fresh leaf material with the NucleoSpin plant kit (Macherey-Nagel; Düren, Germany). We amplified and sequenced nuclear ribosomal external and internal transcribed spacers (ETS and ITS, respectively), and a fragment of the *Xdh* gene. We also sequenced a ~1500 bp fragment of the plastid gene *ycf*1, as well as the *trn*S–*trn*G intergenic spacer. Amplification and sequencing settings, as well as sequencing primers used for ITS, ETS, *Xdh*, *trn*S–*trn*G, and *ycf*1, are the same as previously reported^64, 81^ (Tab. S2). In this study, 84 sequences were newly generated (Tab. S1).

Loci were aligned separately using MAFFT 7.1^82^. For nuclear ribosomal RNA loci and plastid *trn*S–*trn*G spacer, secondary structure of molecules was taken into account (i.e. the -qINSi option). Congruence between nuclear and plastid datasets was assessed as previously done by Pérez-Escobar and colleagues^64^, and using PACo^83^. The procedure is available as a pipeline (http://www.uv.es/cophylpaco/) and was also employed to identify operational terminal units (OTUs) from the plastid dataset that are in conflict with the nuclear dataset (potential outliers detected by PACo are shown in Figs S1 and S2). A detailed explanation on PACo and a rationale on outlier handling is provided as Extended Materials and Methods of Appendix S1.

Phylogenetic analyses of separate and concatenated loci were carried out under maximum likelihood (ML) and Bayesian inference (BI). The best-fitting evolutionary models for ML and Bayesian analyses (for each data partition) were selected using jModelTest v.2.1.6^84^, relying on a Likelihood Ratio Test (LRT) and the Akaike information criterion (AIC) (Tab. S3). Phylogenetic inference relied on the ML approach implemented in RAXML-HPC v.8.2.4^85^ and BI as implemented in MRBAYES v.3.2.2^86^ and were carried out on the CIPRES Science Gateway computing facility^87^. Bayesian inferences were performed with two independent runs, each with four Markov chains Monte Carlo (MCMC) running for 30 million generations each, and sampled every 1000^th^ generation (all other prior settings by default). Log files derived from MRBAYES were examined, and the convergence of MCMC was assessed using TRACER (available at: http://tree.bio.ed.ac.uk/software/tracer/). Node support values were assessed for both the ML tree using MLBS and the consensus Bayesian tree using PP.

### Molecular clock dating

A few orchid macrofossils are available for Orchidaceae^88, 89^, but these are assigned to lineages very distantly related to our groups of interest. Using distant outgroups to calibrate our *Cycnoches* phylogeny would have created extensive sampling heterogeneities, which can result in spurious results^90^. Thus, we had to rely on secondary calibrations. In order to obtain the best secondary calibration points possible, we first generated an Orchidaceae-wide fossil-calibrated phylogeny, sampling 316 orchid species as representatively as possible along the tree. Loci, number of sequences, and settings for absolute age estimation of the Orchidaceae-wide fossil calibrate phylogeny are provided in the Extended Materials and Methods of Appendix S1. The ages obtained were very similar to recent orchid dating studies^91, 92^, and the dated phylogeny is shown in Figure S3.

We selected two secondary calibrations for dating of *Cycnoches*: (*i*) the crown group of Catasetinae was set to 19.8 Mya with a standard deviation of 4 to reflect the 95% CI, and (*ii*) and the root of the *Cycnoches* tree (i.e. MRCA of *Cyrtopodium* + Catasetinae) was set to 27.1 Mya with a standard deviation of 6. To explore the clock-likeness of the data, we used both strict clock and uncorrelated lognormal clock models, and compared different tree priors (pure-birth, standard birth-death, and incomplete sampling birth-death). For strict molecular clock calibration, we placed a single constraint only at the tree root (27.1 Mya with a standard deviation of 6) using a normal distribution. The best-fitting tree speciation model was selected using Bayes factors calculated from marginal likelihoods computed for every model using the stepping-stone sampling^93^ (Tab. 1). For each clock model, we ran two MCMC analyses with 20 million generations each, sampled every 1000^th^ generation. For the relaxed molecular clock analyses, we estimated the coefficient of variation (CV) to inform us on the rate heterogeneity among branches (CV approaching zero indicates that a strict clock model cannot be rejected). Parameter convergence was confirmed using TRACER (http://tree.bio.ed.ac.uk/software/tracer/). All dating analyses were performed at the CIPRES Science Gateway computing facility^87^.

### Ancestral area estimations

Species ranges of *Cycnoches* were coded from the collection site and/or type locality of the material sequenced, which reflect the distribution ranges for every taxon included in our phylogeny (except for *C*. *chlorochilon* and *C*. *pentadactylon*, which also occur in Central America and Southeastern South America, respectively; see below). Representative taxa from *Catasetum*, *Cyrtopodium*, *Galeandra*, and *Mormodes* (~36% of the total species sampling - 17 species) that cover the distribution range of *Cycnoches*, were chosen as outgroup for biogeographical analyses. Coding matrix for ingroup and outgroup taxa is provided in Appendix S1. Distribution data of *Cycnoches* and outgroup taxa were obtained from own field observations, literature^94, 95^ and from herbarium specimens (Herbaria AMES, COL, F, M, MO, SEL, US); this information was employed to code outgroup distribution ranges.

Biogeographical areas were derived from distribution maps of the orchids under investigation as well as species distributions observed in other plant lineages (e.g. Rubiaceae^6^; Bromeliaceae^43^). We coded for eight biogeographical areas using the R-package ‘SpeciesGeocodeR’^96^: (1) Central America comprising southern Mexico through Panama; (2) Caribbean-Llanos comprising the coastal northernmost areas and plains of Colombia and Venezuela^97^; (3) Guiana Shield encompasses areas above 800 m in Colombia, Venezuela, Brazil, Guyana, Suriname, and French Guiana; (4) Amazonia encompassing lowlands and pre-montane forest below 800 m in Colombia, Ecuador, Peru, Brazil, Venezuela, Guyana, Suriname, and French Guiana^6^; (5) Chocó comprising lowlands below 500 m of the western Andes in Colombia and Ecuador; (6) Northern Andes including elevated areas above 800 m from Southernmost Peru to Northern Colombia and Northeast Venezuela; (7) Central Andes comprising areas above 800 m in Northern Peru to Northern Chile and Northeast Argentina; (8) South-eastern South America encompassing part of the Brazilian shield, the Atlantic forest, South-eastern Bolivia, Paraguay, Uruguay, and Northern Argentina.

To infer ancestral areas in *Cycnoches*, we used the R-package ‘BioGeoBEARS’ (Biogeography with Bayesian and Likelihood Evolutionary Analysis in R script^98^). Using BioGeoBEARS, we tested the fit of six biogeographic models with and without founder-event speciation (or jump speciation), altogether testing the role and contribution of evolutionary processes that were taken into account to explain today’s observed distributions (i.e. range expansions, local extinctions, founder-event speciation, vicariance, and speciation despite sympatry) in a joint statistical framework. It is therefore capable of model testing and of determining, which process fits better the geographical and phylogenetic data for any particular clade.

## Electronic supplementary material

Appendix S1

## References

[1] Humboldt, A. *Voyage aux regions equinoxiales du Nouveau Continent*. (N. Mazé, 1820).

[2] Darwin, C. *Geological observations of South America*. (Smith, Elder and CO., 1846).

[3] Jaramillo C, Rueda MJ, Mora G (2006). Cenozoic plant diversity in the Neotropics. Science.

[4] Antonelli A, Sanmartín I (2011). Why are there so many plant species in the Neotropics?. Taxon.

[5] Myers N, Mittermeier RA, Mittermeier CG, Fonseca GAB, Kent J (2000). Biodiversity hotspots for conservation priorities. Nature.

[6] Antonelli A, Nylander JAA, Persson C, Sanmartín I (2009). Tracing the impact of the Andean uplift on Neotropical plant evolution. Proc. Natl. Acad. Sci. USA.

[7] Hoorn C (2010). Amazonia through time: Andean uplift, climate change, landscape evolution, and biodiversity. Science.

[8] Bacon CD (2015). Biological evidence supports an early and complex emergence of the Isthmus of Panama. Proc. Natl. Acad. Sci. USA.

[9] Lagomarsino L, Condamine FL, Antonelli A, Mulch A, Davis CC (2016). The abiotic and biotic drivers of rapid diversification in Andean bellflowers (Campanulaceae). New Phytol..

[10] Hughes CE, Pennington RT, Antonelli A (2013). Neotropical plant evolution: assembling the big picture. Bot. J. Linn. Soc..

[11] Moonlight PW (2015). Continental-scale diversification patterns in a megadiverse genus: the biogeography of Neotropical Begonia. J. Biogeogr..

[12] Pennington RT (2010). Contrasting plant diversification histories within the Andean biodiversity hotspot. Proc. Natl. Acad. Sci. USA.

[13] Viruel J (2016). Late Cretaceous-Early Eocene origin of yams (*Dioscorea*, Dioscoreaceae) in the Laurasian Palaearctic and their subsequent Oligocene-Miocene diversification. J. Biogeogr..

[14] Luebert F, Hilger HH, Weigend M (2011). Diversification in the Andes: age and origins of South American *Heliotropium* lineages (Heliotropiaceae, Boraginales). Mol. Phylogenet. Evol..

[15] Hughes C, Eastwood R (2006). Island radiation on a continental scale: exceptional rates of plant diversification after uplift of the Andes. Proc. Natl. Acad. Sci. USA.

[16] Nevado B, Atchison GW, Hughes CE, Filatov DA (2016). Widespread adaptive evolution during repeated evolutionary radiations in New World lupins. Nat. Commun..

[17] Bacon CD, Mora A, Wagner WL, Jaramillo CA (2013). Testing geological models of evolution of the Isthmus of Panama in a phylogenetic framework. Bot. J. Linn. Soc..

[18] Pérez-Escobar, O. A. *et al*. Recent origin and rapid speciation of Neotropical orchids in the world’s richest plant biodiversity hotspot. *New Phytol*. **215**, 891–905 (2017). 10.1111/nph.14629 PMC5575461 28631324

[19] Luebert F, Weigend M (2014). Phylogenetic insights into Andean plant diversification. Front. Ecol. Evol..

[20] van der Hammen T (1974). The Pleistocene changes of vegetation and climate in Tropical South America. J. Biogeogr..

[21] Martínez C, Madriñán S, Zavada M, Jaramillo CA (2013). Tracing the fossil pollen record of *Hedyosmum* (Chloranthaceae), an old lineage with recent Neotropical diversification. Grana.

[22] Ghosh P, Garzione CN, Eiler JM (2006). Rapid uplift of the Altiplano revealed through 13C-18O bonds in Paleosol carbonates. Science.

[23] Kar N (2016). Rapid regional surface uplift of the northern Altiplano plateau revealed by multiproxy paleoclimate reconstruction. Earth Planet. Sci. Lett..

[24] Hoorn C, Guerrero J, Sarmiento GA, Lorente MA (1995). Andean tectonics as a cause for changing drainage patterns in Miocene northern South America. Geology.

[25] Gregory-Wodzicki KM (2000). Uplift history of the Central and Northern Andes: a review. Geol. Soc. Am. Bull.

[26] Armijo R, Lacassin R, Coudurier-Curveur A, Carrizo D (2015). Coupled tectonic evolution of Andean orogeny and global climate. Earth Sci. Rev..

[27] Gentry AH (1982). Neotropical floristic diversity: phytogeographical connections between Central and South America, Pleistocene climatic fluctuations, or an accident of the Andean orogeny?. Ann. Missouri Bot. Gard..

[28] Richter M, Diertl K, Emck P, Peters T, Beck E (2009). Reasons for an outstanding plant diversity in the tropical Andes of Southern Ecuador. Landsc. Online.

[29] Pirie MD, Chatrou LW, Mols JB, Erkens RHJ, Oosterhof J (2006). ‘Andean-centred’ genera in the short-branch clade of Annonaceae: testing biogeographical hypotheses using phylogeny reconstruction and molecular dating. J. Biogeogr..

[30] Janzen DH, Martin PS (1982). Neotropical anachronisms: the fruits the gomphotheres ate. Science.

[31] Uribe-Convers S, Tank DC (2015). Shifts in diversification rates linked to biogeographic movement into new areas: An example of a recent radiation in the Andes. Am. J. Bot..

[32] Sánchez-Baracaldo P (2004). Phylogenetics and biogegraphy of the Neotropical fern genera *Jamesonia* and *Eriosorus* (Pteridaceae). Am. J. Bot..

[33] Kreft H, Koster N, Kuper W, Nieder J, Barthlott W (2004). Diversity and biogeography of vascular epiphytes in Western Amazonia, Yasuni, Ecuador. J. Biogeogr.

[34] Krömer T, Kessler M, Gradstein SR, Acebey A (2005). Diversity patterns of vascular epiphytes along an elevational gradient in the Andes. J. Biogeogr..

[35] Gentry AH, Dodson CH (1987). Diversity and biogeography of Neotropical vascular epiphytes. Ann. Missouri Bot. Gard..

[36] Mutke J, Barthlott W (2005). Patterns of vascular plant diversity at continental to global scales. Biol. Skr..

[37] Kier G (2005). Global patterns of plant diversity and floristic knowledge. J. Biogeogr.

[38] Zotz G (2013). The systematic distribution of vascular epiphytes-a critical update. Bot. J. Linn. Soc..

[39] Nauheimer L, Boyce PC, Renner SS (2012). Giant taro and its relatives: a phylogeny of the large genus *Alocasia* (Araceae) sheds light on Miocene floristic exchange in the Malesian region. Mol. Phylogenet. Evol..

[40] Murren CJ (2003). Spatial and demographic population genetic structure in *Catasetum viridiflavum* across a human-disturbed habitat. J. Evol. Biol..

[41] Murren C, Ellison A (1998). Seed dispersal characteristics of *Brassavola nodosa* (Orchidaceae). Am. J. Bot..

[42] Arditti J, Ghani AKA (2000). Numerical and physical properties of orchid seeds and their biological implications. New Phytol..

[43] Givnish TJ (2011). Phylogeny, adaptive radiation, and historical biogeography in Bromeliaceae: insights from an eight-locus plastid phylogeny. Am. J. Bot..

[44] Pridgeon, A. M., Cribb, P. J., Chase, M. W. & Rasmussen, F. N. *Genera Orchidacearum: Vol*. *5*. *Epidendroideae* (*part two*). (Oxford University Press, 2009).

[45] Pérez-Escobar OA, Gottschling M, Whitten WM, Salazar G, Gerlach G (2016). Sex and the Catasetinae (Darwin’s favourite orchids). Mol. Phylogenet. Evol..

[46] Ramirez SR (2011). Asynchronous diversification in a specialized plant-pollinator mutualism. Science.

[47] Chase MW, Pippen JS (1990). Seed morphology and phylogeny in Subtribe Catasetinae (Orchidaceae). Lindleyana.

[48] Romero GA (1990). Phylogenetic relationships in Subtribe Catasetinae (Orchidaceae, Cymbidieae). Lindleyana.

[49] Stern W, Judd W (2001). Comparative anatomy and systematics of Catasetinae (Orchidaceae). Bot. J. Linn. Soc..

[50] Batista JN (2014). Molecular phylogenetics of Neotropical *Cyanaeorchis* (Cymbidieae, Epidendroideae, Orchidaceae): geographical rather than morphological similarities plus a new specie. Phytotaxa.

[51] Whitten WM, Neubig KM, Williams NH (2014). Generic and subtribal relationships in neotropical Cymbidieae (Orchidaceae) based on *matK/ycf1* plastid data. Lankesteriana.

[52] Carr, G. F. J. The genus *Cycnoches*: species and hybrids. *Orchid Rev*. 1–31 (2012).

[53] Gerlach G, Pérez-Escobar OA (2014). Looking for missins swans: phylogenetics of *Cycnoches*. Orchids.

[54] Darwin, C. *On the various contrivances by which british and foreign orchids are fertilised by insects*. (Appleton and CO., 1877).

[55] Pridgeon, A. M. & Chase, M. W. Phylogenetics of subtribe Catasetinae (Orchidaceae) from nuclear and chloroplast DNA sequences. In *Proceeding of the 15th World Orchid Conference* 275–281 (1998).

[56] Romero, G. A. & Gerlach, G. *Cycnoches*. In *Flora Mesoamericana* (Missouri Botanical Garden Press).

[57] Garzione CN, Molnar P, Libarkin JC, Macfadden BJ (2006). Rapid late Miocene rise of the Bolivian Altiplano: evidence for removal of mantle lithosphere. Earth Planet. Sci. Lett..

[58] Garzione CN (2008). Rise of the Andes. Nature.

[59] Garzione CN (2014). Clumped isotope evidence for diachronous surface cooling of the Altiplano and pulsed surface uplift of the Central Andes. Earth Planet. Sci. Lett..

[60] Alves RV, Kolbek J (1994). Plant species endemism in savanna vegetation on table mountains (Campo Rupestre) in Brazil. Vegetatio.

[61] Sánchez-Baracaldo P, Thomas GH (2014). Adaptation and convergent evolution within the *Jamesonia-Eriosorus* complex in high-elevation biodiverse andean hotspots. PLoS One.

[62] Jersáková J, Malinová T (2004). Spatial aspects of seed dispersal and seedling recruitment in orchids. New Phytol..

[63] Barthlott, W., Große-Veldmann, B. & Korotkova, N. *Orchid seed diversity: a scanning electron microscopy survey*. (Botanic Garden and Botanical Museum Berlin, 2014).

[64] Pérez-Escobar OA, Balbuena JA, Gottschling M (2016). Rumbling orchids: how to assess divergent evolution between chloroplast endosymbionts and the nuclear host. Syst. Biol..

[65] Rokas A, Williams BL, King N, Carroll SB (2003). Genome-scale approaches to resolving incongruence in molecular phylogenies. Nature.

[66] Salichos L, Stamatakis A, Rokas A (2014). Novel information theory-based measures for quantifying incongruence among phylogenetic trees. Mol. Biol. Evol..

[67] Petit RJ, Excoffier L (2009). Gene flow and species delimitation. Trends Ecol. Evol..

[68] van der Niet T, Peter Linder H (2008). Dealing with incongruence in the quest for the species tree: a case study from the orchid genus *Satyrium*. Mol. Phylogenet. Evol..

[69] Vargas, O. M., Ortiz, E. M. & Simpson, B. B. Conflicting phylogenomic signals reveal a pattern of reticulate evolution in a recent high‐Andean diversification (Asteraceae: Astereae: *Diplostephium*). *New Phytol*., doi:10.1111/NPH.14530 (2017). 10.1111/nph.14530 28333396

[70] Rieseberg LH, Beckstrom-Sternberg S, Doan K (1990). Helianthus annuus ssp. texanus has chloroplast DNA and nuclear ribosomal RNA genes of Helianthus debilis ssp. cucumerifolius. Proc. Natl. Acad. Sci. USA.

[71] Rieseberg LH, Soltis DE (1991). Phylogenetic consequences of cytoplasmatic gene flow in plants. Evol. Trends Plants.

[72] Wolf PG, Murray RA, Sipes SD (1997). Species-independent, geographical structuring of chloroplast DNA haplotypes in a montane herb *Ipomopsis* (Polemoniaceae). Mol. Ecol..

[73] Rieseberg LH, Whitton J, Linder CR (1996). Molecular marker incongruence in plant hybrid zones and phylogenetic trees. Acta Bot. Neerl..

[74] Chang S-B, Chen W-H, Chen H-H, Fu Y-M, Lin Y-S (2000). RFLP and inheritance patterns of chloroplast DNA in intergenic hybrids of *Phalaenopsis* and *Doritis*. Bot. Stud..

[75] Cafasso D, Widmer A, Cozzolino S (2005). Chloroplast DNA inheritance in the orchid Anacamptis palustris using single-seed polymerase chain reaction. J. Hered..

[76] Petit RJ (2005). Comparative organization of chloroplast, mitochondrial and nuclear diversity in plant populations. Mol. Ecol..

[77] Gerlach G, Schill R (1991). Composition of orchid scents attracting Euglossine bees. Bot. Acta.

[78] Dressler RL (1967). Pollination by Euglossine bees. Evolution.

[79] Williams H, Whitten WM (1983). Orchid floral fragrances and male euglossine bees: methods and advances in the last sesquidecade. Biol. Bull..

[80] Gregg KB (1983). Variation in floral fragrances and morphology: incipient speciation in *Cycnoches*?. Bot. Gaz..

[81] Irimia R-E, A. Pérez-Escobar O, Gottschling M (2014). Strong biogeographic signal in the phylogenetic relationships of *Rochefortia* Sw. (Ehretiaceae, Boraginales). Plant Syst. Evol..

[82] Katoh K, Standley DM (2013). MAFFT multiple sequence alignment software version 7: improvements in performance and usability. Mol. Biol. Evol..

[83] Balbuena JA, Míguez-Lozano R, Blasco-Costa I (2013). PACo: a novel procrustes application to cophylogenetic analysis. PLoS One.

[84] Darriba D, Taboada GL, Doallo R, Posada D (2012). jModelTest 2: more models, new heuristics and parallel computing. Nat. Methods.

[85] Stamatakis A (2014). RAxML version 8: a tool for phylogenetic analysis and post-analysis of large phylogenies. Bioinformatics.

[86] Ronquist F (2012). Mrbayes 3.2: Efficient Bayesian phylogenetic inference and model choice across a large model space. Syst. Biol..

[87] Miller MA (2015). A RESTful API for access to phylogenetic tools via the CIPRES Science Gateway. Evol. Bioinforma..

[88] Ramírez SR, Gravendeel B, Singer RB, Marshall CR, Pierce NE (2007). Dating the origin of the Orchidaceae from a fossil orchid with its pollinator. Nature.

[89] Conran JG, Bannister JM, Lee DE (2009). Earliest orchid macrofossils: early Miocene *Dendrobium* and *Earina* (Orchidaceae: Epidendroideae) from New Zealand. Am. J. Bot..

[90] Drummond, A. J. & Bouckaert, R. R. *Bayesian evolutionary analysis with BEAST 2*. (Cambridge University Press, 2014).

[91] Chomicki G (2015). The velamen protects photosynthetic orchid roots against UV-B damage, and a large dated phylogeny implies multiple gains and losses of this function during the Cenozoic. New Phytol..

[92] Givnish TJ (2016). Orchid historical biogeography, diversification, Antarctica and the paradox of orchid dispersal. J. Biogeogr..

[93] Xie W, Lewis PO, Fan Y, Kuo L, Chen MH (2011). Improving marginal likelihood estimation for bayesian phylogenetic model selection. Syst. Biol..

[94] Carr, G. F. J. The genus *Cycnoches* and its species Part 4: modern studies of pollinators. *Orchid Rev*. 221–225 (2006).

[95] Romero, G. A. Cymidieae. In *Genera Orchidacearum: Vol*. *5*. Epidendroideae *(part two)* (eds Pridgeon, A. M., Cribb, P. J., Chase, M. W. & Rasmussen, F. N.) 11–40 (Oxford University Press, 2009).

[96] Töpel M (2014). SpeciesGeoCoder: Fast categorisation of species occurrences for analyses of biodiversity, biogeography, ecology and evolution. BioRxiv.

[97] Morrone JJ (2006). Biogeographic areas and transition zones of Latin America and the Caribbean islands based on panbiogeographic and cladistic analyses of the entomofauna. Annu. Rev. Entomol..

[98] Matzke NJ (2014). Model selection in historical biogeography reveals that founder-event speciation is a crucial process in island clades. Syst. Biol..

