# Supplementary material for: Andean Mountain Building Did not Preclude Dispersal of Lowland Epiphytic Orchids in the Neotropics
Source: Sci Rep. 2017 Jul 7;7:4919. doi: 10.1038/s41598-017-04261-z (PMC5501825; doi:10.1038/s41598-017-04261-z)
Supplement: Supplementary file 1 — Appendix S1 [file 41598_2017_4261_MOESM1_ESM.pdf]

ANDEAN MOUNTAIN BUILDING DID NOT PRECLUDE DISPERSAL OF LOWLAND EPIPHYTIC ORCHIDS IN THE NEOTROPICS

Oscar Alejandro Pérez-Escobar*, Marc Gottschling, Guillaume Chomicki, Fabien L. Condamine, Bente Klitgård, Emerson Pansarin and Günter Gerlach*

Appendix S1

Including: Extended material and methods, Tables S1-S6, Figures S1-S6.

Including: coding matrix for biogeographical analysis (https://github.com/siriusb-nox/Andean_uplift_orchid_dispersal.git)

Extended materials and methods

Phylogenetic incongruence analysis

PACo builds upon co-phylogenetic approaches to assess the similarities between two trees by comparison of Euclidean distance matrices derived from phylogenies[1]. Additionally, it determines the contribution of every association (i.e., pair of terminals of the analysed phylogenies) to the co-phylogenetic pattern observed thus, it has the potential to statistically identify specific potential conflicting terminals. PACo requires as input data a set of Posterior Probability or Likelihood Bootstrap phylograms derived from every genomic compartment (i.e., 'nuc' and 'cp') and a binary association matrix, in which same taxa present in both datasets are linked. We executed PACo using 1000 Bayesian Posterior Probabilities obtained from Mr.Bayes analysis computed from concatenated nuclear and plastid datasets, respectively.

Plastids are maternally inherited in orchids[2,3] and hence, they reflect maternal evolutionary relationships between species only. In contrast, nuclear loci show bi-parental inheritance [4] thus revealing maternal and paternal evolutionary histories. Our nuclear derived phylogeny strongly reflects morphological relationships, whereas the plastid tree revealed an apparent geographical pattern (see *Results* and *Discussion*). Following these principles, 20 conflicting plastid sequences belonging to eleven species of *Cycnoches* and one of *Dressleria* were excluded from the dataset (Table S6), and non-conflicting sequences of each locus were re-aligned and concatenated.

Orchidaceae phylogeny molecular clock dating

We compiled 264 sequences from nuclear ITS locus, 297 sequences from *mat*K, 276 from *rbc*L, and 217 from *trn*L-*trn*F (Tab. S4). Molecular clock dating relied on the uncorrelated lognormal relaxed clock model implemented in BEAST v.2.0[5]. We used the

GTR + G nucleotide substitution model with 6 rate categories, a Yule tree prior, and a Markov chain Monte Carlo (MCMC) chain length of 100 million generations, sampling every 10000 generations. Four fossil calibrations were used. The oldest known fossil of the Asparagales (105 Mya[6]) was used as a maximal calibration point for the Orchidaceae following[7] and was given a normal prior (offset = 105.3; SD = 8). A gamma distribution was used on each of the three ingroup fossil constraints (the offset value was set to the minimum age of each fossil and the standard deviation was set such that the maximum age was included in the 97.5% quantile). The fossil orchid †*Meliochis caribea* (15–20 Mya) has been assigned to the Goodyerinae[8], and provided a minimum age for the subtribe (offset = 15; SD= 5). Two recently discovered orchid macrofossils (†*Dendrobium winkaphyllum* and †*Earina fouldenensis*, both 20–23 Mya[9], were used as minimum constraints within Epidendroideae, with gamma priors (offset = 20; SD = 4.5). The trees were summarized using TREEANNOTATOR v.1.8.0 (included in the BEAST package), with a 10% burn-in and a 0.98 posterior probability limit.

Ancestral area estimations

Twenty-tree out of the 34 accepted species of *Cycnoches* were sampled in this study. Missing species not included in the sampling are distributed in Amazonia region and because of their morphological features, are probably nested in clade C, together with all the other species distributed in the Amazon (i.e., *C. manoelae, C. peruvinaum, C. quatuorcristis,* and *C. suarezii*). Therefore, it is likely that they will have little effect on the ancestral area estimation analyses performed.

References

1. Balbuena, J. A., Míguez-Lozano, R. & Blasco-Costa, I. PACo: a novel procrustes application to cophylogenetic analysis. *PLoS One* **8,** e61048 (2013).
2. Chang, S.-B., Chen, W.-H., Chen, H.-H., Fu, Y.-M. & Lin, Y.-S. RFLP and inheritance patterns of chloroplast DNA in intergenic hybrids of Phalaenopsis and Doritis. *Bot. Stud.* **41,** 219–223 (2000).
3. Cafasso, D., Widmer, A. & Cozzolino, S. Chloroplast DNA inheritance in the orchid Anacamptis palustris using single-seed polymerase chain reaction. *J. Hered.* **96,** 66–70 (2005).
4. Petit, R. J. *et al.* Comparative organization of chloroplast , mitochondrial and nuclear diversity in plant populations. *Mol. Ecol.* **14,** 689–701 (2005).
5. Bouckaert, R. *et al.* BEAST 2: a software platform for Bayesian evolutionary analysis. *PLoS Comput. Biol.* **10,** 1–6 (2014).
6. Walker, J. W. & Walker, A. G. Ultrastructure of lower Cretaceous angiosperm pollen and early evolution of flowering plants. *Ann. Missouri Bot. Gard.* **71,** 464–521 (1984).
7. Gustafsson, A. L. S., Verola, C. F. & Antonelli, A. Reassessing the temporal evolution of orchids with new fossils and a Bayesian relaxed clock , with implications for the diversification of the rare South American genus Hoffmannseggella (Orchidaceae : Epidendroideae). *BMC Evol. Biol.* **10,** 177–190 (2010).
8. Ramírez, S. R., Gravendeel, B., Singer, R. B., Marshall, C. R. & Pierce, N. E. Dating the origin of the Orchidaceae from a fossil orchid with its pollinator. *Nature* **448,** 1042–1045 (2007).
9. Conran, J. G., Bannister, J. M. & Lee, D. E. Earliest orchid macrofossils: Early Miocene Dendrobium and Earina (Orchidaceae: Epidendroideae) from New Zealand. *Am. J. Bot.* **96,** 466–474 (2009).

10. Baldwin, B. G. Phylogenetic utility of the internal transcribed spacers of nuclear ribosomal DNA in plants: An example from the compositae. *Mol. Phylogenet. Evol.* **1,** 3–16 (1992).

11. Monteiro, S. H. N., Selbach-Schnadelbach, A., de Oliveira, R. P. & van den Berg, C. Molecular Phylogenetics of <I>Galeandra</I> (Orchidaceae: Catasetinae) based on Plastid and Nuclear DNA Sequences. *Syst. Bot.* **35,** 476–486 (2010).

12. Baldwin, B. G. & Markos, S. Phylogenetic utility of the external transcribed spacer (ETS) of 18S-26S rDNA: congruence of ETS and ITS trees of Calycadenia (Compositae). *Mol. Phylogenet. Evol.* **10,** 449–63 (1998).

13. Górniak, M., Paun, O. & Chase, M. W. Phylogenetic relationships within Orchidaceae based on a low-copy nuclear coding gene, Xdh: Congruence with organellar and nuclear ribosomal DNA results. *Mol. Phylogenet. Evol.* **56,** 784–795 (2010).

14. Neubig, K. M. *et al.* Phylogenetic utility of ycf1 in orchids: a plastid gene more variable than matK. *Plant Syst. Evol.* **277,** 75–84 (2008).

15. Hamilton, M. B. Primer Notes: Four primer pairs for the amplification of chloroplast intergenic regions with intraspecific variation. *Mol. Ecol.* **8,** 513–525 (1999).

Supplementary tables

Table S1. Species names and voucher information, including herbarium specimens of voucher deposition, for material used in this study to build *Cycnoches* molecular dataset. Taxa sequenced for this study are shown in boldface.

Table S2. Primers and PCR settings used for amplifying chloroplast and nuclear DNA loci.

Table S3. Evolutionary models used for phylogenetic reconstructions, as selected by the software jModelTest.

Table S4. Species names and voucher information, including herbarium specimens of voucher deposition, for material used in this study to build Orchidaceae molecular dataset.

Table S5. Alignment characterization.

Table S6. Potential conflicting Operational Terminal Units (OTUs) between nuclear and plastid phylogenies detected by PACo. Squared residual values (E_i^2) are provided (cut-off value of 0.0204). Terminals and species highlighted in green are true outliers.

Supplementary figures

Figure S1. Conflicting nuclear-plastid associations (i.e., pair of nuclear / plastid taxa) obtained by PACo analysis, using posterior probability trees. Taxa with normalized squared residual values above the cut-off threshold (red line; see accessions with red bars) indicate potential conflicting associations. Blue whiskers indicate 95% empirical confidence intervals.

Figure S2. Phylogenetic relationships of *Cycnoches* independently derived 59 from nuclear (ITS, ETS, *Xdh*) and plastid loci (*trn*S–*trn*G, *ycf*1) datasets. Potential outlier, congruent and excluded terminals (i.e. taxa with sequences included on the plastid loci but missing on the nuclear dataset prior analysis) are shown in red, green and blue, respectively.

Figure S3. Chronogram for Orchidaceae obtained under a relaxed clock model, applied to a non-conflicting, concatenated nuclear ITS *Xdh* and plastid *mat*K, *rbc*L loci. Mean absolute ages and 95% Confidence Intervals are shown. The MRCA of Catasetinae is labelled with a red star.

Figure S4. Chronogram for *Cycnoches* obtained under a relaxed clock model, applied to a non-conflicting, concatenated nuclear (ITS, ETS, *Xdh*) and plastid (*trn*S–*trn*G, *ycf*1) loci. Mean absolute ages and 95% Confidence Intervals are shown.

Figure S5. Probabilities of ancestral areas estimated from a chronogram of *Cycnoches* and the BayArea-like model including the founding speciation process. Geographical distribution in coded biogeographical areas of sampled species is shown in front of tree terminals. (Inset) Coded areas used for biogeographical analysis. Geopolitical boundaries map generated by ArcMAP software (http://www.esri.com). Political divisions and elevation data from DIVA-GIS (http://www.diva-gis.org/gdata).

Table S1

Species	Voucher	ITS	ETS	*Xdh*	*Trn*S-G	*ycf1*
Cycnoches amparoanum Schltr.	DNA43 (M)	**MF285471**	**MF285449**	**MF285517**	**MF285507**	**MF285533**
Cycnoches aureum Lindl. & Paxton	DNA57 (M)	KT768390	KT768355	KT768459	KT768426	KT768496
	DNA62 (M)	**MF285472**	**MF285450**	-	-	**MF285534**
Cycnoches barthiorum G.F.Carr & Christenson	DNA5 (M)	KT768391	KT768356	KT768460	KT768427	KT768497
Cycnoches chlorochilon Klotzsch	DNA152 (M)	**MF285473**	-	-	-	-
	DNA32 (M)	KT768392	KT768357	KT768461	KT768428	KT768498
Cycnoches cooperi Rolfe	DNA161 (M)	**MF285474**	-	-	-	-
	DNA68 (M)	KT768393	KT768358	KT768462	KT768429	-
Cycnoches densiflorum Rolfe	DNA6 (M)	KT768394	KT768359	KT768463	KT768430	**MF285535**
	DNA65 (M)	**MF285475**	**MF285451**	-	-	**MF285536**
Cycnoches dianae Rchb. f.	DNA36 (M)	**MF285476**	**MF285452**	-	**MF285508**	**MF285537**
	DNA52 (M)	KT768395	KT768360	KT768464	-	KT768501
	DNA54 (M)	**MF285477**	**MF285453**	**MF285518**	-	**MF285538**
Cycnoches egertonianum Bateman	DNA145 (M)	**MF285482**	-	-	-	-
	DNA146 (M)	**MF285485**	-	-	-	-
	DNA147 (M)	**MF285483**	-	-	-	-
	DNA150 (M)	**MF285484**	-	-	-	-
	DNA22 (M)	**MF285478**	**MF285454**	-	-	**MF285539**
	DNA31 (M)	KT768396	KT768361	KT768465	KT768433	-
	DNA40 (M)	**MF285479**	**MF285455**	**MF285519**	KT768432	KT768502
	DNA47 (M)	**MF285480**	**MF285456**	-	-	**MF285540**
	DNA48 (M)	**MF285481**	-	-	-	-
	DNA99 (M)	-	**MF285457**	**MF285520**	**MF285509**	**MF285541**
Cycnoches guttulatum Schltr.	DNA128 (M)	**MF285486**	**MF285458**	**MF285521**	-	**MF285542**
	DNA35 (M)	**MF285487**	**MF285459**	-	-	**MF285543**
	DNA58 (M)	KT768398	KT768363	KT768467	KT768434	KT768504

Cycnoches haagii Barb. Rodr.	DNA37 (M)	KU257702	KU257695	KU257718	KU257710	KU257726
	DNA8 (M)	**MF285488**	**MF285460**	**MF285522**	KT768435	KT768505
	DNA95 (M)	**MF285489**	**MF285461**	-	-	**MF285544**
Cycnoches herrenhusanum Jenny & G.A. Romero	DNA100 (M)	KU257703	KU257696	KU257719	KU257711	KU257727
	DNA11 (M)	**MF285490**	**MF285462**	KT768469	KT768436	KT768506
Cycnoches lehmannii Rchb. f.	DNA1 (M)	KT768401	KT768366	KT768470	KT768437	KT768507
	DNA72 (M)	**MF285491**	**MF285463**	-	-	**MF285545**
Cycnoches loddigesii Lindl.	DNA10 (M)	KT768402	KT768367	KT768471	KT768438	KT768508
	DNA96 (M)	**MF285492**	**MF285464**	**MF285523**	-	-
Cycnoches maculatum Lindl.	DNA153 (M)	**MF285493**	-	-	-	-
Cycnoches manoelae V.P. Castro & Campacci	DNA38 (M)	KT768403	KT768368	KT768472	KT768439	KT768509
	DNA67 (M)	**MF285494**	-	-	-	-
Cycnoches pachydactylon Schltr.	DNA53 (M)	KT768404	KT768369	KT768473	KT768440	KT768510
	DNA55 (M)	**MF285495**	-	-	-	-
	DNA66 (M)	**MF285496**	-	-	-	-
Cycnoches pentadactylon Lindl.	DNA24 (M)	KU257704	KT768370	-	-	-
	DNA34 (M)	**MF285497**	**MF285465**	-	-	**MF285546**
Cycnoches peruvianum Rolfe	DNA123 (M)	**MF285498**	**MF285466**	**MF285524**	**MF285510**	**MF285547**
	DNA15 (M)	**MF285499**	-	**MF285525**	KT768443	KT768513
	DNA3 (M)	KT768405	KT768371	KU257720	-	**MF285548**
Cycnoches quatuorcristis D.E.Benn.	DNA69 (M)	**MF285500**	**MF285467**	-	-	-
Cycnoches rossianum Rolfe	DNA141 (M)	**MF285501**	-	-	-	-
	DNA50 (M)	**MF285502**	**MF285468**	**MF285526**	**MF285511**	**MF285549**
Cycnoches suarezii Dodson	DNA39 (M)	KT768408	KT768374	KT768476	KT768444	KT768515
Cycnoches ventricosum Bateman	DNA4 (M)	KT768409	KT768375	KT768477	-	**MF285550**
	DNA41 (M)	**MF285503**	**MF285469**	-	**MF285512**	**MF285551**
Cycnoches warszewiczii Rchb. f.	DNA21 (M)	**MF285504**	**MF285470**	**MF285527**	**MF285513**	**MF285552**
	DNA9 (M)	KT768410	KT768376	KT768478	KT768446	KT768517
**OUTGROUP**						

Catasetum collare Cogn.	DNA16 (M)	KT768384	KT768350	KT768454	-	KT768421
Catasetum juruenense Hoehne	DNA77 (M)	KT768385	KT768351	KT768455	-	KT768422
Cyrtopodium andersonii (Lamb. ex Andrews) R. Br.	DNA130 (M)	-	-	**MF285528**	-	**MF285553**
Cyrtopodium eugenii Rchb.f. & Warm.	DNA131 (M)	-	-	**MF285529**	-	-
Cyrtopodium gigas (Vell.) Hoehne	DNA132 (M)	-	-	**MF285530**	-	-
Cyrtopodium glutiniferum Raddi	Pansarin-sn (SPF)	-	-	-	**MF285514**	**MF285554**
Cyrtopodium paludicola Hoehne	DNA133 (M)	-	-	**MF285531**	-	-
Cyrtopodium punctatum (L.) Lindl.	DNA 118 (M)	-	-	-	-	-
Cyrtopodium saintlegerianum Rchb.f.	DNA134 (M)	-	-	**MF285532**	-	KY988621
Dressleria severiniana H.G.Hills	DNA104 (M)	**MF285505**	-	KU257721	-	KU257729
Eulophia petersii (Rchb.f.) Rchb.f.	DNA108 (M)	**MF285506**	-	KT768481	**MF285515**	**MF285555**
Galeandra leptoceras Schltr.	DNA105 (M)	KU257706	KU257699	KU257722	**MF285516**	**MF285556**
Mormodes badia Rolfe ex W.Watson	DNA76 (M)	KT768415	KT768380	KT768484	KU257715	KU257732
Mormodes ephippilabia Fowlie	DNA26 (M)	KT768416	KT768381	KT768485	-	KT768526
Mormodes luxata Lindl.	DNA17 (M)	KT768417	KT768382	KT768486	-	KU257733
Mormodes punctata Rolfe	DNA75 (M)	KT768418	KT768383	KT768487	KU257716	KT768528
Mormodes tigrina Barb. Rodr.	DNA106 (M)	KU257707	KU257700	KU257723	KU257717	**MF285557**

Table S2

Loci	Primer	Sequence	Reference	Pre-melt	Amplification	Final extension	Number of amplification cycles
ITS	ITS 4	TCC-TCC-GCT-TAT-TGA-TAT-GC	Baldwin[10]	95°C (3 min)	95°C (30 secs) + 52°C (1 min) + 68°C (1 min)	68°C (10 min)	39
	ITS 5	GGA-AGT-AAA-AGT-CGT-AAC-AAG-G		95°C (3 min)	95°C (30 secs) + 52°C (1 min) + 68°C (1 min)	68°C (10 min)	39
ETS	EST-Orchid	CAT-ATG-AGT-TGT-TGC-GGA-CC (AT)-T	Monteiro et al.[11]	95°C (3 min)	95°C (30 secs) + 52°C (1 min) + 68°C (1 min)	68°C (10 min)	39
	18-IGS	AGA-CAA-GCA-TAT-GAC-TAC-TGG-CAG-G	Balwin and Markos[12]	95°C (3 min)	95°C (30 secs) + 52°C (1 min) + 68°C (1 min)	68°C (10 min)	39
Xdh	X502F	TGT-GAT-GTC-GAT-GTA-TGC	Górniak et al.[13]	95°C (3 min)	95°C (30 secs) + 53°C (1 min) + 68°C (1.5 min)	68°C (10 min)	39
	X1599R	G(AT)G-AGA-GAA-A(CT)TG-GAG-CAA-C		95°C (3 min)	95°C (30 secs) + 53°C (1 min) + 68°C (1.5 min)	68°C (10 min)	39
Ycf1	3720F	TAC-GTA-TGT-AAT-GAA-CGA-ATG-G	Neubig et al.[14]	95°C (3 min)	95°C (30 secs) + 54°C (1 min) + 68°C (1.5 min)	68°C (10 min)	39
	5500R	GCT-GTT-ATT-GGC-ATC-AAA-CCA-ATA-GCG		95°C (3 min)	95°C (30 secs) + 54°C (1 min) + 68°C (1.5 min)	68°C (10 min)	39
*trn*S-G	trn-S(GCU)	GCC-GCT-TTA-GTC-CAC-TCA-GC	Hamilton[15]	95°C (3 min)	95°C (30 secs) + 51.5°C (1 min) + 68°C (1.5 min)	68°C (10 min)	39
	trn-G(UCC)	GAA-CGA-ATC-ACA-CTT-TTA-CCA-C		95°C (3 min)	95°C (30 secs) + 51.5°C (1 min) + 68°C (1.5 min)	68°C (10 min)	39

Table S3

**Locus**	**Model**	**Log Likelihood**	**AIC**
ITS	GTR+G	-3322,82933	6887,658
ETS	TPM3uf+G	-2047,147	4298,295
Xdh	HKY+G	-2359,67223	4881,34
*Trn*S-G	TPM1uf+G	-1888,024	3908,04
*ycf*1	TVM+G	-4072,666	8349,33

Table S4

Species	matK	rbcL	ITS	trnL-trnF
Aa paleacea (Kunth) Rchb.f.	AJ309989.1	AF074105	FJ473308.1	FJ571262.1
Acanthophippium mantinianum L.Linden & Cogn.	AF263618.1	AF074100.1	AF521081.1	AF519966.1
*Acianthus exsertus*R.Br.	AJ309993.1	AF074101.1	-	AJ409373.1
Acineta superba(Kunth) Rchb.f.	AF239475.1	-	AF239379.1	AF239571.1
Acriopsis liliifolia (J.König) Seidenf.	AF470462.1	-	AF470492.1	-
Brassia euodes Rchb.f.	FJ564987.1	FJ534215.1	FJ565503.1	FJ562390.1
Agrostophyllum majus Hook.f.	AY368391.1	AF518054.1	AF521080.1	AF519964.1
Altensteinia fimbriata Kunth	AM900801.1	FJ571315.1	AM419765.1	FJ571265.1
Epidendrum armeniacum Lindl.	AF263748	AF518058	AF260165	
Galearis rotundifolia (Banks ex Pursh) R.M.Bateman	JN966073.1	AY149368.1	JN998910.1	GQ244552.1
Amitostigma monanthum (Finet) Schltr.	JN696436.1	JN696421.1	-	-
Anacamptis pyramidalis (L.) Rich.	JN895643.1	JN892999.1	AY364870.1	-
Ancistrochilus rothschildianus O'Brien	AY121729.1	AF264152.1	AF521061.1	AF519928.1
Ansellia africana O'Brien	AF470461.1	EU213445.1	-	-
Anthogonium gracile Wall. ex Lindl.	AF263622.1	AF264153.1	AF273336.1	AF519930.1
Aplectrum hyemale (Muhl. ex Willd.) Nutt.	EF525693.1	EU391356.1	AY008468.1	EU266434.1
Aporostylis bifolia (Hook.f.) Rupp & Hatch	-	AY381109.1	AF347980.1	-
Apostasia nuda R.Br.	AY557214.1	-	AY557230.1	KC172528.1
Appendicula cornuta Blume	AY121739.1	AF518050.1	AF521073.1	AF519956.1
Arethusa bulbosa L.	AF263624.1	AF264154.1	AF273338.1	AF519912.1
Arpophyllum giganteum Hartw. ex Lindl.	AF265485.1	AF074110	AF266742.1	AF265527.1
Arundina graminifolia (D.Don) Hochr.	AF302692.1	AF074111.1	JN114439.1	AF519931.1
Aspasia lunata Lindl.	EF079200.1	FJ534160.1	FJ565252.1	FJ562360.1
Astelia alpina R.Br.	AY368372.1	Z77261	-	HM459530.1
Aulosepalum nelsonii (Greenm.) Garay	AM884246.1	-	AM884878.1	-
Basiphyllaea hamiltoniana Ackerman & Whitten	AY121721.1	AF518026.1	AF521050.1	AF519903.1

Baskervilla colombiana Garay	AM900826.1	AM778157.1	AM419791.1	FJ571266.1
Beloglottis costaricensis (Rchb.f.) Schltr.	AJ543920.1	AJ542432.1	AJ539492.2	AJ544475.1
Bifrenaria harrisoniae (Hook.) Rchb.f.	AY368394.1	AF074112	-	AY063435.1
Bipinnula montana Arechav.	FR832065.1	FR831959.1	FR832102.1	FR832137.1
Bletia purpurea (Lam.) DC.	AF302693.1	AF518022.1	AF273337.1	AY008451.1
Bletilla striata (Thunb.) Rchb.f.	KC704596.1	AF074114.1	AF461466.1	AF519939.1
Bonatea speciosa (L.f.) Willd.	EU214217.1	EU213446.1	DQ522070.1	-
Brassia andina (Rchb.f.) M.W.Chase	FJ565088.1	FJ534236.1	FJ565599.1	FJ562402.1
Broughtonia sanguinea (Sw.) R.Br.	AF263790.1	AF518069.1	AF260186.1	AF267015.1
Brownleea macroceras Sond.	DQ414995.1	-	DQ414852.1	GU117880.1
Bulbophyllum macranthum Lindl.	JF305768.1	D58405.1	EF195933.1	JF428047.1
Caladenia flava R.Br.	GQ866546.1	-	GQ866490.1	GQ866809.1
Calanthe discolor Lindl.	HM640665.1	AF264159	-	-
Calopogon tuberosus (L.) Britton, Sterns & Poggenb.	AF263635.1	AF074119.1	AF273395.1	-
Calypso bulbosa (L.) Oakes	EF525689.1	AF074120.1	AF521076.1	AF519960.1
Camaridium nutantiflorum Schltr.	AF239427.1	AF074190	DQ210450.1	AF239523.1
Capanemia superflua (Rchb.f.) Garay	FJ563840.1	FJ534139.1	FJ565195.1	FJ562353.1
Catasetum expansum Rchb.f.	AF263637.1	AF074121.1	-	-
Cattleya dowiana Bateman & Rchb.f.	AF263638.1	AF074122	AY008593.1	AF267045.1
Caucaea phalaenopsis (Linden & Rchb.f.) N.H.Williams & M.W.Chase	FJ565012.1	FJ534221.1	FJ565529.1	FJ562394.1
Caularthron bilamellatum (Rchb.f.) R.E.Schult.	AF263780.1	AF518059.1	AF260173.1	AF267001.1
Cephalanthera humilis X. H. Jin	JN706692.1	JN706688.1	-	JN706694.1
Ceratostylis rubra Ames	AY368397.1	AY368353.1	-	-
Chamaegastrodia shikokiana Makino & F.Maek.	KC704643.1	JN166038.1	JN166061.1	-
Chiloglottis trapeziformis Fitzg.	AJ310003.1	AF074124	AY042153.1	AY042121.1
Chloraea piquichen (Lam.) Lindl.	GQ917025.1	-	JQ045501.1	-
Christensonella pacholskii (Christenson) S.Koehler	DQ210889.1	-	DQ210402.1	EU099788.1
Chysis bractescens Lindl.	EF079351.1	AF074126.1	EF079363.1	AF266971.1

Chytroglossa marileoniae Rchb.f.	FJ565112.1	FJ534244.1	DQ315817.1	FJ562408.1
Cischweinfia dasyandra (Rchb.f.) Dressler & N.H.Williams	FJ565125.1	FJ534248.1	FJ565633.1	FJ562412.1
Cleistes rosea Lindl.	AJ310006.1	AF074128	-	AJ409385.1
Clematepistephium smilacifolium (Rchb.f.) N.Hallé	-	AF074131.1	FJ425838.1	-
Coccineorchis cernua (Lindl.) Garay	AJ543930.1	AJ542422.1	AJ539502.1	AJ544485.1
Codonorchis lessonii (d'Urv.) Lindl.	DQ414993.1	AY381113.1	AF348005.1	-
Coelia triptera (Sm.) G.Don ex Steud	AF263643.1	AF074132.1	EF079362.1	AF266972.1
Coelogyne cristata Lindl.	AF302707.1	AF074133.1	JN114444.1	AF519937.1
Coelogyne flaccida Lindl.	AF302708.1	KC797588.1	AF029855.1	-
Coilochilus neocaledonicum Schltr.	AJ310009.1	AY381114.1	AF348006.1	AJ409388.1
Collabium simplex Rchb.f.	AY557200.1	-	EF670387.1	EF670434.1
Comparettia falcata Poepp. & Endl.	FJ565090.1	FJ534237.1	FJ565601.1	FJ562403.1
Corallorhiza odontorhiza (Willd.) Nutt.	EF525701.1	EU391360.1	EU391326.1	-
Coryanthes macrantha (Hook.) Hook.	AF239455.1	-	AF239359.1	AF239551.1
Corybas diemenicus (Lindl.) Rupp	AF263646.1	AF074135.1	AF391769.1	AJ409389.1
Corycium carnosum (Lindl.) Rolfe	EU301524.1	AY381115.1	EU301471.1	EU301577.1
Cranichis ciliilabia C.Schweinf.	AJ543934.1	AJ542419.1	AF391781.1	-
Cremastra appendiculata (D.Don) Makino	EF525691.1	EU391354.1	EU266414.1	EU266439.1
Cryptarrhena lunata R.Br.	AF239420.1	-	AF239324.1	AF239516.1
Cryptocentrum peruvianum (Cogn.) C.Schweinf.	DQ210786.1	AF074139.1	DQ210321.1	-
Cryptostylis subulata (Labill.) Rchb.f.	AJ310015.1	AF074140.1	AF348015.1	AJ409395.1
Cuitlauzina candida (Lindl.) Dressler & N.H.Williams	FJ563853.1	FJ534253.1	FJ565219.1	FJ562416.1
Cyanicula gemmata (Lindl.) Hopper & A.P.Br.	GQ866553.1	AY381116.1	-	-
Cyclopogon peruvianus (C.Presl) Schltr.	HE575496.1	FJ571323.1	HE575513.1	HE575523.1
Cycnoches egertonianum Bateman	AY368401.1	AY368355.1	-	-
Cymbidium ensifolium (L.) Sw.	AF263648.1	AF074141	AF284716.1	-
Cypripedium passerinum Richardson	AF263649.1	AF074142	JN999131.1	JF796858.1
Cyrtidiorchis stumpflei (Garay) Rauschert	FJ564741.1	-	FJ565229.1	-
Cyrtopodium andersonii (Lamb. ex Andrews) R.Br.	AF470460.1	AF074143.1	AF470490.1	-

Cystorchis aphylla Ridl.	JN166021.1	JN166039.1	JN166062.1	-
Dactylorhiza fuchsii (Druce) Soó	JN895597.1	JQ933294	DQ022864.1	AY284953.1
Dendrobium albopurpureum (Seidenf.) Schuit. & Peter B.Adams	AF448865.1	FJ216574.1	-	-
Dendrobium amplum Lindl. In N.Wallich	KF143738.1	KF177669.1	KF143529.1	KF143635.1
Dendrobium catenatum Lindl.	KF143649.1	KF177581.1	KF143438.1	KF143546.1
Dendrobium crystallinum Rchb.f.	KF143657.1	KF177590.1	HQ114243.1	EF397917.1
Dendrobium kingianum Bidwill ex Lindl.	AF263651.1	AF074146.1	EU430386.1	AF519958.1
Dendrobium nobile Lindl.	KF143702.1	AB519785	EF618732.1	KF143600.1
Dendrochilum glumaceum Lindl.	AF302696.1	AF264164.1	AF461465.1	AF519933.1
Dendrophylax lindenii (Lindl.) Benth. ex Rolfe	AF506362.1	-	AF506318.1	AF506338.1
Dichaea panamensis Lindl.	EU123650.1	-	EU123584.1	EU123709.1
Dichromanthus cinnabarinus (Lex.) Garay	AJ543914.1	AJ542438.1	FN996952.1	FN996964.1
Cuitlauzina pygmaea (Lindl.) M.W.Chase & N.H.Williams	FR832752.1	FN870802.1	-	-
Dilomilis montana (Sw.) Summerh.	AF263765.1	AF074150.1	AF262915.1	AF266967.1
Dinema polybulbon (Sw.) Lindl.	AF263769.1	AF518061.1	AF260154.1	AY422413.1
Disa glandulosa Burch. ex Lindl.	AF263654.1	AF274006	DQ414873.1	DQ415158.1
Disperis lindleyana Rchb.f.	AY370652.1	AY370651	AJ000129.1	EU301601.1
Diuris sulphurea R.Br.	AF263655.1	AF074152	DQ904018.1	AY851053.1
Dossinia marmorata C.Morren	AJ543947.1	AJ542405	JN166065.1	-
Dracula chimaera (Rchb.f.) Luer	AF265444.1	AF518039.1	AF262766.1	AF265489.1
Dressleria dilecta (Rchb.f.) Dodson	EF079265.1	-	AF239411.1	-
Duckeella adolphii Porto & Brade	FR832756.1	EU498134.1	EU498159.1	EU498219.1
Earina autumnalis (G.Forst.) Hook.f.	AF263656.1	AF074155.1	AF260149.1	KF591259.1
Earina valida Rchb.f.	AY121741.1	AF518051.1	AF521077.1	AY008448.1
Eleorchis japonica (A.Gray) Maek.	AF263657.1	AF264166.1	AF521055.1	AF519914.1
Elleanthus purpureus (Rchb.f.) Rchb.f.	HQ328988.1	-	HM854581.1	-
Eltroplectris triloba (Lindl.) Pabst	FN868835.1	-	FN641864.1	FN641875.1
Empodium veratrifolium (Willd.) M.F.Thomps.	AY368374.1	Y14987.1	-	HM459497.1

Encyclia cordigera (Kunth) Dressler	AY396114.1	-	AY008528.1	AY422417.1
Entomophobia kinabaluensis (Ames) de Vogel	AF302697.1	AF518036.1	AF461464.1	AF519938.1
Epipactis helleborine (L.) Crantz	AF263659.1	Z73707.1	AY154383.1	AB428735.1
Epistephium subrepens Hoehne	-	AF074163.1	FJ425837.1	-
Eria ferruginea Lindl.	AF263660.1	AF074164	AF521071.1	AF519954.1
Eriaxis rigida Rchb.f.	-	AF074165.1	FJ425833.1	-
Eriochilus cucullatus (Labill.) Rchb.f.	AJ310028.1	AF074166	AF348030.1	AJ409410.1
Eriopsis biloba Lindl.	DQ461806.1	AF074167.1	DQ461788.1	-
Erycina glossomystax (Rchb.f.) N.H.Williams & M.W.Chase	FJ565076.1	FJ534231.1	FJ565589.1	FJ562398.1
Erythrorchis cassythoides (A.Cunn. ex Lindl.) Garay	-	AF074169.1	FJ425841.1	-
Eulophia petersii (Rchb.f.) Rchb.f.	AF263661.1	AF264167.1	-	-
Eulophiella elisabethae Linden & Rolfe	FR832765.1	FN870819.1	-	-
Ponthieva fertilis (F.Lehm. & Kraenzl.) Salazar	AJ310013.1	FJ571322.1	FJ473318.1	FJ571272.1
Fernandezia ionanthera (Rchb.f. & Warsz.) Schltr.	FJ565010.1	FJ534219.1	FJ565527.1	AF239582.1
Funkiella hyemalis (A.Rich. & Galeotti) Schltr.	AJ543923.1	AJ542429.1	AJ539495.1	AJ544478.1
Galeandra devoniana M.R.Schomb. ex Lindl.	AY368408.1	AF074171	EU877142.1	-
Gastrochilus retrocallus (Hayata) Hayata	AB217731.1	-	AB217555.1	DQ194986.1
Gavilea venosa (Lam.) Garay & Ormerod	JQ045542.1	FR831991.1	FR832132.1	FR832165.1
Gennaria diphylla (Link) Parl.	AY368383.1	AY368341.1	AY351380.1	-
Genoplesium fimbriatum (R.Br.) D.L.Jones & M.A.Clem.	AJ310031.1	JQ933453.1	-	AJ409413.1
Geodorum densiflorum (Lam.) Schltr.	FR832768.1	JQ933343.1	JN114512.1	-
Glomera hamadryas (Schltr.) J.J.Sm.	-	AF518053.1	AF521079.1	AF519963.1
Glossodia major R.Br.	GQ866560.1	AF074173.1	GQ866508.1	GQ866825.1
Gomesa flexuosa (Lodd.) M.W.Chase & N.H.Williams	FJ565141.1	FJ534252.1	FJ565649.1	FJ562415.1
Gomphichis caucana Schltr.	AM900805.1	AM778136.1	AM419770.1	AM412736.1
Gonatostylis vieillardii (Rchb.f.) Schltr.	GQ917045.1	FJ571328.1	FJ473325.1	FJ571278.1
Gongora amparoana Schltr.	AY368409.1	AY368358	AF239385.1	AF239577.1

Goodyera pubescens (Willd.) R.Br.	AF263663.1	AF074174	FJ473326.1	FJ571279.1
Govenia liliacea (Lex.) Lindl.	AY121723.1	-	AF521056.1	AF519916.1
Grammatophyllum speciosum Blume	AF239510.1	AF074176.1	AF470488.1	AF239606.1
Graphorkis lurida (Sw.) Kuntze	AY368410.1	AY368359.1	-	-
Grobya galeata Lindl.	AF470457.1	AY370655.1	AF470487.1	-
Gymnadenia conopsea (L.) R.Br.	EF612530.1	KC704906.1	JF414046.1	AF105322.1
Gynoglottis cymbidioides (Rchb.f.) J.J.Sm.	FR832773.1	FN870833.1	-	-
Habenaria repens Nutt.	AJ310036.1	AF074177	HM777628.1	AJ409418.1
Trichopilia sanguinolenta (Lindl.) Rchb.f	FJ564986.1	FJ534214.1	FJ565502.1	AF350738.1
Helleriella guerrerensis Dressler & Hágsater	AF263761.1	AF518029.1	AF260142.1	AF266961.1
Hemipilia calophylla E.C.Parish & Rchb.f.	JN696444.1	JN696429.1	-	-
Herminium lanceum (Thunb. ex Sw.) Vuikj	JN696440.1	JN696425.1	JN114589.1	-
Hetaeria elata Hook.f.	-	JN166048.1	JN166068.1	-
Hexalectris revoluta Correll	AF263665.1	AF264168.1	FJ457858.1	FJ427741.1
Himantoglossum robertianbum (Loisel.) P.Delforge	AY368382.1	AY368337.1	AY351384.1	AY014584.1
Pterostemma benzingii (Dodson) M.W.Chase & N.H.Williams	FJ565102.1	FJ534242.1	FJ565612.1	-
Hofmeisterella eumicroscopica (Rchb.f.) Rchb.f.	FJ565091.1	FJ534238.1	DQ315823.1	AF350668.1
Holcoglossum sinicum Christenson	EU558956.1	HQ404496.1	HQ452906.1	HQ452936.1
Houlletia sanderi Rolfe	AF239467.1	AF074178.1	AF239371.1	AF239563.1
Huntleya wallisii (Rchb.f.) Rolfe	EU123674.1	-	AY870075.1	AY869887.1
Hygrochilus parishii (Veitch & Rchb.f.) Pfitzer	AB217733.1	JN005546.1	EF079439.1	-
Hylophila lanceolata (Blume) Miq.	JN166030.1	JN166051.1	JN166070.1	-
Hypoxis curtissii Rose	AY368375.1	Z73702	-	HM459504.1
Ionopsis utricularioides (Sw.) Lindl.	FJ565042.1	FJ534225.1	FJ565557.1	FJ562395.1
Isochilus chiriquensis Schltr.	AF263762.1	AY368361.1	AF260143.1	AF266962.1

Isotria verticillata (Muehl. ex Willd.) Raf.	-	EU498135.1	EU498160.1	EU498226.1
Kegeliella kupperi Mansf.	AF263666.1	AF074181.1	AF239364.1	AF239556.1
Koellensteinia graminea (Lindl.) Rchb.f.	AY870003.1	AF074182.1	AY870102.1	AY869906.1
Kreodanthus simplex (C.Schweinf.) Garay	-	FJ571331.1	FJ473328.1	FJ571281.1
Kuhlhasseltia nakaiana (F.Maek.) Ormerod	JN166031.1	JN166052.1	JN166071.1	-
Laelia speciosa (Kunth) Schltr.	AF263792.1	AF518070	AY008578.1	AF267018.1
Lanaria lanata (L.) T.Durand & Schinz	AY368376.1	Z77313	-	HM459536.1
Leochilus carinatus (Knowles & Westc.) Lindl.	FJ564709.1	FJ534138.1	FJ565193.1	FJ562352.1
Lepanthes woodburyana Stimson	AF265472.1	-	AF262890.1	AF265494.1
Lepidogyne longifolia (Blume) Blume	JN166032.1	JN166053.1	JN166072.1	-
Leporella fimbriata (Lindl.) A.S.George	AJ310038.1	AY381124.1	AF348038.1	AJ409420.1
Lockhartia bennettii Dodson	FJ565011.1	FJ534220.1	FJ565528.1	FJ562393.1
Ludisia discolor (Ker Gawl.) A.Rich.	AJ543911.1	AJ542395	JN166073.1	-
Luisia tristis (G.Forst.) Hook.f.	JN004493.1	JN005554.1	JN114606.1	-
Lycaste cruenta (Lindl.) Lindl.	AF239438.1	AF074185.1	AF239342.1	AF239534.1
Manniella cypripedioides Salazar, T.Franke, Zapfack & Beenken	AJ543943.1	AJ542409.1	AJ539516.1	AJ544498.1
Masdevallia floribunda Lindl.	AY368416.1	AF074189	AY008475.1	AF266966.1
Maxillaria porrecta Lindl.	DQ211043.1	-	DQ210568.1	-
Meiracyllium trinasutum Rchb.f.	AF263670.1	AF074192.1	AY429390.1	AY422393.1
Mesadenus polyanthus (Rchb.f.) Schltr.	AM902109.1	FJ571317.1	FJ473313.1	FJ571267.1
Mexipedium xerophyticum (Soto Arenas, Salazar & Hágsater) V.A.Albert & M.W.Chase	JN181455.1	AF074193.1	FR720330.1	FR851215.1
Microchilus arietinus (Rchb.f. & Warm.) Ormerod	-	FJ571324.1	FJ473320.1	FJ571274.1
Microtis unifolia (G.Forst.) Rchb.f.	AJ310045.1	AF074194	DQ104553.1	AJ409428.1
Miltonia regnellii Rchb.f.	AF239491.1	FJ534193.1	EF079409.1	AF239587.1
Mormodes aromatica Lindl.	AY368417.1	AF074196	-	-
Mormolyca ringens (Lindl.) Gentil	DQ210680.1	AY368363.1	DQ210219.1	-
Myrosmodes cochleare Garay	-	FJ571333.1	FJ473330.1	FJ571283.1

Neocogniauxia hexaptera (Cogn.) Schltr.	AF263766.1	AF518037.1	AY429385.1	AY422444.1
Neolindleya camtschatica (Cham.) Nevski	KC704641.1	KC704904.1	-	-
Neomoorea wallisii (Rchb.f.) Schltr.	DQ210743.1	-	AF239341.1	AF239533.1
Neottia smallii (Wiegand) Szalch.	AF263668.1	AF074184	AF521058.1	AF519920.1
Nephelaphyllum pulchrum Blume	-	AF518049.1	AF521070.1	AF519952.1
Nervilia shirensis (Rolfe) Schltr.	AY368420.1	-	AF521066.1	AF519945.1
Neuwiedia veratrifolia Blume	AY557211.1	AF074200	AY557227.1	KC172538.1
Notyliopsis beatricis P.Ortiz	FJ565086.1	FJ534234.1	FJ565597.1	FJ562401.1
Octomeria gracilis Lodd. ex Lindl.	AF265484.1	AF518044.1	AF262911.1	AF265526.1
Oncidium strictum (Cogn.) M.W.Chase & N.H.Williams	FJ564985.1	FJ534145.1	FJ565501.1	-
Odontorrhynchus variabilis	AJ543926.1	AJ542426.1	AF348047.1	AJ544481.1
Oeceoclades maculata (Lindl.) Lindl.	JQ588555.1	JQ593044.1	-	-
Vitekorchis excavata (Lindl.) Romowicz & Szlach.	AY368423.1	AF074201	FJ565604.1	FJ562405.1
Ophrys apifera Huds.	AJ543953.1	AF074202.1	AM711790.1	AJ409432.1
Orchis quadripunctata Cirillo ex Ten.	AY368385.1	AF074203	-	-
Oreorchis patens (Lindl.) Lindl.	EF525694.1	EU391355.1	JN252495.1	EU266436.1
Ornithocephalus dressleri (Toscano) Toscano & Dressler	FJ565083.1	FJ534233.1	FJ565594.1	FJ562400.1
Orthoceras strictum R.Br.	AJ310050.1	AF074204.1	AF348048.1	AJ409433.1
Otochilus lancilabius Seidenf.	AY299364.1	-	HQ130500.1	-
Otoglossum coronarium (Lindl.) Garay & Dunst.	FJ565100.1	FJ534241.1	FJ565610.1	FJ562406.1
Fernandezia crystallina (Lindl.) M.W.Chase	FJ565077.1	FJ534232.1	FJ565590.1	FJ562399.1
Pachyplectron arifolium Schltr.	AJ310051.1	AF074205	FJ473334.1	AJ409434.1
Palmorchis trilobulata L.O.Williams	AJ310052.1	AF074206	-	AJ409435.1
Paphiopedilum delenatii Guillaumin	AY368379.1	AF074208	JQ660881.1	-
Papilionanthe teres (Roxb.) Schltr.	HQ439021.1	HQ439016.1	DQ091682.1	EU558872.1
Parapteroceras quisumbingii (L.O.Williams) J.J.Wood	FR832805.1	FN870890.1	-	-
Pelatantheria scolopendrifolia (Makino) Aver.	AB217722.1	-	AB217546.1	-
Pelexia laxa (Poepp. & Endl.) Lindl.	-	FJ571337.1	FJ473335.1	FJ571287.1
Phaius flavus (Blume) Lindl.	AF263676.1	AF074210.1	AF521051.1	AF519907.1

Phalaenopsis equestris (Schauer) Rchb.f.	AF263677.1	AF074211	AY912225.1	AY273651.1
Pholidota articulata Lindl.	JF422080.1	JN005633.1	HM590390.1	-
Pholidota pallida Lindl.	JF422081.1	JN005637.1	-	-
Phragmipedium schlimii (Linden ex Rchb.f.) Rolfe	AY368380.1	AF074213	JQ929360.1	JF796884.1
Phreatia tahitensis Lindl.	AY368425.1	-	AF521065.1	AF519944.1
Physoceras boryanum (A.Rich.) Bosser	FR832811.1	FN870897.1	-	-
Pilophyllum villosum (Blume) Schltr.	FR832812.1	FN870898.1	-	-
Pityphyllum antioquiense Schltr.	DQ209961.1	-	DQ210371.1	-
Platanthera ciliaris (L.) Lindl.	AF263678.1	AF074215	-	-
Platanthera uniformis Tang & F.T.Wang	JN696434.1	JN696419.1	JN696448.1	-
Platanthera urceolata (Hook.f.) R.M.Bateman	FR832753.1	JQ933305.1	JN696447.1	-
Platylepis polyadenia Rchb.f.	AJ543946.1	AJ542406.1	AJ539520.1	AJ544501.1
Platythelys querceticola (Lindl.) Garay	AY368386.1	AF074216	FJ473336.1	FJ571288.1
Plectorrhiza tridentata (Lindl.) Dockrill	FR832813.1	FN870901.1	-	-
Plectrophora cultrifolia (Barb.Rodr.) Cogn.	FJ564979.1	FJ534213.1	FJ565495.1	-
Pleione formosana Hayata	AF263679.1	AF264173	AF461485.1	AF503686.1
Pleurothallis ruscifolia (Jacq.) R.Br.	AF265463.1	AF518042.1	JF934813.1	AF265500.1
Pleurothallopsis nemorosa (Barb.Rodr.) Porto & Brade	AF291104.1	FN870902.1	AF291099.1	AF291100.1
Podochilus cultratus Lindl.	AY121738.1	AF074218.1	AF521072.1	AF519955.1
Pogonia ophioglossoides (L.) Ker Gawl.	AJ310055.1	AF074221	EU498161.1	EU498225.1
Polystachya neobenthamia Schltr.	AY121734.1	AF518045.1	GU556663.1	DQ091434.1
Polystachya pubescens (Lindl.) Rchb.f.	AY368426.1	AF074222.1	HM018554.1	-
Ponera striata Lindl.	AF263764.1	AF518034.1	AY008486.1	AY422380.1
Ponthieva racemosa (Walter) C.Mohr	AJ310056.1	AF074223	-	AJ409439.1
Porphyrostachys pilifera (Kunth) Rchb.f.	AJ543942.1	FJ571341.1	FJ473339.1	FJ571291.1
Prescottia stachyodes (Sw.) Lindl.	AM900808.1	FJ571342.1	AM419773.1	FJ571292.1
Pristiglottis montana (Schltr.) Cretz. & J.J.Sm.	AJ310061.1	FJ571345.1	JQ045488.1	FJ571295.1

Promenaea ovatiloba (Klinge) Cogn.	AY368428.1	AY368369.1	AY870100.1	AY869904.1
Prosthechea abbreviata (Schltr.) W.E.Higgins	AF263757.1	AF518063.1	AF260181.1	AF267010.1
Galeoglossum thysanochilum (B.L.Rob. & Greenm.) Salazar	AM900810.1	AM778141.1	AM419775.1	AM412725.1
Pseudolaelia vellozicola (Hoehne) Porto & Brade	AF263776.1	AF518057.1	EF079384.1	AF266994.1
Pseudovanilla ponapensis (Kaneh. & Yamam.) Garay	-	AY381131.1	-	-
Psychopsis sanderae (Rolfe) Lückel & Braem	FJ564712.1	FJ534141.1	FJ565198.1	FJ562354.1
Pterichis triloba (Lindl.) Schltr.	AM900830.1	FJ571347.1	AM419795.1	FJ571297.1
Pterostylis longifolia R.Br.	AJ310062.1	-	AY134639.1	AJ409445.1
Pterygodium catholicum (L.) Sw.	FJ469844.1	AY368346.1	FJ469901.1	FJ469867.1
Fernandezia ecuadorensis (Dodson) M.W.Chase	FJ565127.1	FJ534249.1	FJ565635.1	FJ562413.1
Renanthera imschootiana Rolfe	JN004604.1	JN005670.1	JN114705.1	GU185932.1
Renanthera matutina (Poir.) Lindl.	FR832822.1	FN870918.1	AY912263.1	AY273688.1
Restrepia muscifera (Lindl.) Rchb.f. ex Lindl.	AY370654.1	AY370653.1	AF262908.1	-
Rhodohypoxis milloides (Baker) Hilliard & B.L.Burtt	AY368377.1	Z77280	-	-
Rhynchostele cordata (Lindl.) Soto Arenas & Salazar	AM889745.1	GQ248690.1	FJ565653.1	-
Rhynchostylis gigantea (Lindl.) Ridl.	AY557202.1	-	GQ251304.1	EF670411.1
Ridleyella paniculata (Ridl.) Schltr.	AY121737.1	AF518048.1	AF521069.1	AF519951.1
Rimacola elliptica (R.Br.) Rupp	AJ310066.1	AY381133.1	AF348060.1	AJ409449.1
Robiquetia bertholdii (Rchb.f.) Schltr.	FR832824.1	FN870920.1	-	-
Rossioglossum ampliatum (Lindl.) M.W.Chase & N.H.Williams	FJ564764.1	FJ534135.1	EF065707.1	FJ562349.1
Rudolfiella floribunda (Schltr.) Hoehne	AF239433.1	-	AF239337.1	AY869912.1
Sacoila lanceolata (Aubl.) Garay	AJ543933.1	AJ542441.1	EU384878.1	AJ544529.1
Sarcoglottis acaulis (Sm.) Schltr.	AJ310068.1	AY368347	FJ473349.1	FJ571300.1
Sauroglossum aurantiacum (C.Schweinf.) Garay	-	FJ571353.1	FJ473352.1	FJ571303.1
Scaphosepalum gibberosum (Rchb.f.) Rolfe	AF265458.1	AF518041.1	AF262817.1	AF265503.1
Scaphyglottis crurigera (Bateman ex Lindl.) Ames & Correll	AF263785.1	AF518065.1	AY174727.1	AF267009.1
Scaphyglottis prolifera (R.Br.) Cogn.	JQ588561.1	JQ593056.1	AY174719.1	-

Schiedeella faucisanguinea (Dod) Burns-Bal. ex A.E.Serna & López-Ferr.	AJ543924.1	AJ542428.1	AJ539496.1	AJ544479.1
Schizochilus flexuosus Harv. ex Rolfe	FR832831.1	FN870929.1	-	-
Schoenorchis micrantha Reinw. ex Blume	JN004615.1	JN005681.1	JN114717.1	-
*Selenipedium chica*Rchb.f.	AY368381.1	AF074227	-	-
Sobralia macrantha Lindl.	AF263681.1	AF074228	HM854651.1	-
Spathoglottis plicata Blume	AY368429.1	AF264175.1	-	-
Spiranthes cernua (L.) Rich.	AF263682.1	AF074229	EU384841.1	EU384784.1
Stanhopea tigrina Bateman ex Lindl.	AY368430.1	AF074230	FJ565224.1	AF239544.1
Stenoglottis longifolia Hook.f.	AY368387.1	AY368349.1	AF348065.1	-
Stenoptera acuta Lindl.	-	FJ571360.1	FJ473355.1	FJ571310.1
Stenorrhynchos speciosum (Jacq.) Rich.	AJ543932.1	AJ542420.1	FN996947.1	FN996959.1
Svenkoeltzia congestiflora (L.O.Williams) Burns-Bal.	AJ543921.1	AJ542431.1	AJ539493.1	AJ544476.1
Systeloglossum acuminatum Ames & C.Schweinf.	AF350607.1	FJ534165.1	AF350528.1	AF350686.1
Tainia penangiana Hook.f.	AF263683.1	AF264176.1	-	-
Telipogon obovatus Lindl.	FJ565093.1	FJ534239.1	FJ565603.1	FJ562404.1
Thaia saprophytica Seidenf.	JN706690.1	JN706687.1	FJ454872.1	-
Thelasis carinata Blume	AY121736.1	AF518047.1	-	AF519950.1
Thelymitra ixioides Sw.	AJ310077.1	-	AY029040.1	AJ409455.1
Thunia alba (Lindl.) Rchb.f.	AF263684.1	AF074233.1	JN114725.1	AF503729.1
Tolumnia gundlachii (C.Wright ex Griseb.) N.H.Williams & Ackerman	FJ565132.1	FJ534251.1	FJ565640.1	-
Trichocentrum lacerum (Lindl.) J.M.H.Shaw	FJ565138.1	FJ534251.1	FJ565646.1	FJ562414.1
Trichoceros antennifer (Humb. & Bonpl.) Kunth	FJ564953.1	FJ534226.1	DQ315883.1	AF350670.1
Trichopilia fragrans (Lindl.) Rchb.f.	FJ565053.1	FJ534229.1	FJ565568.1	-
Trichotosia ferox Blume	AY368432.1	AF074235.1	-	-
Trigonidium egertonianum Bateman ex Lindl.	DQ210740.1	-	DQ210184.1	AF239521.1
Triphora trianthophora (Sw.) Rydb.	AY368433.1	AF074236.1	-	-

Tropidia effusa Rchb.f.	AJ310078.1	-	-	AJ409456.1
Vanda nana L. Gardiner	AB217709.1	JX876897.1	AB217533.1	-
Vanda ampullacea (Roxb.) L. Gardiner	EU558935.1	JQ933227.1	EF079434.1	JX202718.1
Vanda falcata (Thunb.) Beer	AF263673.1	AF074197.1	DQ091684.1	EF670421.1
Vanda pumila Hook.f.	EU558941.1	JQ180387.1	KC244657.1	KC244665.1
Vanda sanderiana (Rchb.f) Rchb.f	FR832764.1	FN870818.1	-	-
Vandopsis gigantea (Lindl.) Pfitzer	EF655805.1	JQ933519.1	EF670376.1	EF670417.1
Vanilla planifolia Jacks. ex Andrews	AF263687.1	AF074242	AF391786.1	AY557223.1
Wullschlaegelia aphylla (Sw.) Rchb.f.	AY368434.1	AY368436	-	-
Xerorchis amazonica Schltr.	AF263688.1	AF074244.1	-	-
Xylobium leontoglossum (Rchb.f.) Benth. ex Rolfe	DQ209939.1	-	DQ210254.1	AF239532.1
Zygopetalum maculatum (Kunth) Garay	AF263689.1	AF074246	AY870097.1	AY869901.1
Zygopetalum maxillare Lodd.	EU123676.1	-	AY870095.1	AY869899.1
Zygostates apiculata (Lindl.) Toscano	FJ565111.1	FJ534243.1	FJ565619.1	FJ562407.1

Table S5

	**ETS**	**ITS**	***Xdh***	**trn*S-G***	***ycf1***
Number of cells	49/56	56/56	39/56	31/56	47/56
Alingment lenght (bp)	544	848	1004	792	1642
Parsimony Informative Sites (no/%)	123/23%	140/16%	49/5%	80/10%	114/7%

Table S6

Conflicting OTU	Species	E_i^2
Cycnoches chlorochilon DNA32	*Cycnoches chlorochilon*	0,026832
Cycnoches cooperi DNA68	*Cycnoches cooperi*	0,020621
Cycnoches densiflorum DNA6	*Cycnoches densiflorum*	0,033645
Cycnoches densiflorum DNA65		0,033836
Cycnoches haagii DNA37	*Cycnoches haagii*	0,027757
Cycnoches haagii DNA8		0,027592
Cycnoches haagii DNA95		0,024298
Cycnoches lehmannii DNA72	*Cycnoches lehmannii*	0,023160
Cycnoches loddigesii DNA10	*Cycnoches loddigesii*	0,026835
Cycnoches manoelae DNA38	*Cycnoches manoelae*	0,025062
Cycnoches pentadactylon DNA34	*Cycnoches pentadactylon*	0,025617
Cycnoches peruvianum DNA123	*Cycnoches peruvianum*	0,028516
Cycnoches peruvianum DNA15		0,026893
Cycnoches peruvianum DNA3		0,027664
Cycnoches suarezii DNA39	*Cycnoches suarezii*	0,024456
Cycnoches ventricosum DNA4	*Cycnoches ventricosum*	0,025247
Cycnoches ventricosum DNA41		0,022342
Cycnoches warszewiczii DNA21	*Cycnoches warszewiczii*	0,025909
Cycnoches warszewiczii DNA9		0,027141
Dressleria severiniana DNA104	*Dressleria severiniana*	0,030862
Mormodes luxata DNA17	*Mormodes luxata*	0,023170
Mormodes tigrina DNA106	*Mormodes tigrina*	0,021489

Figure S1

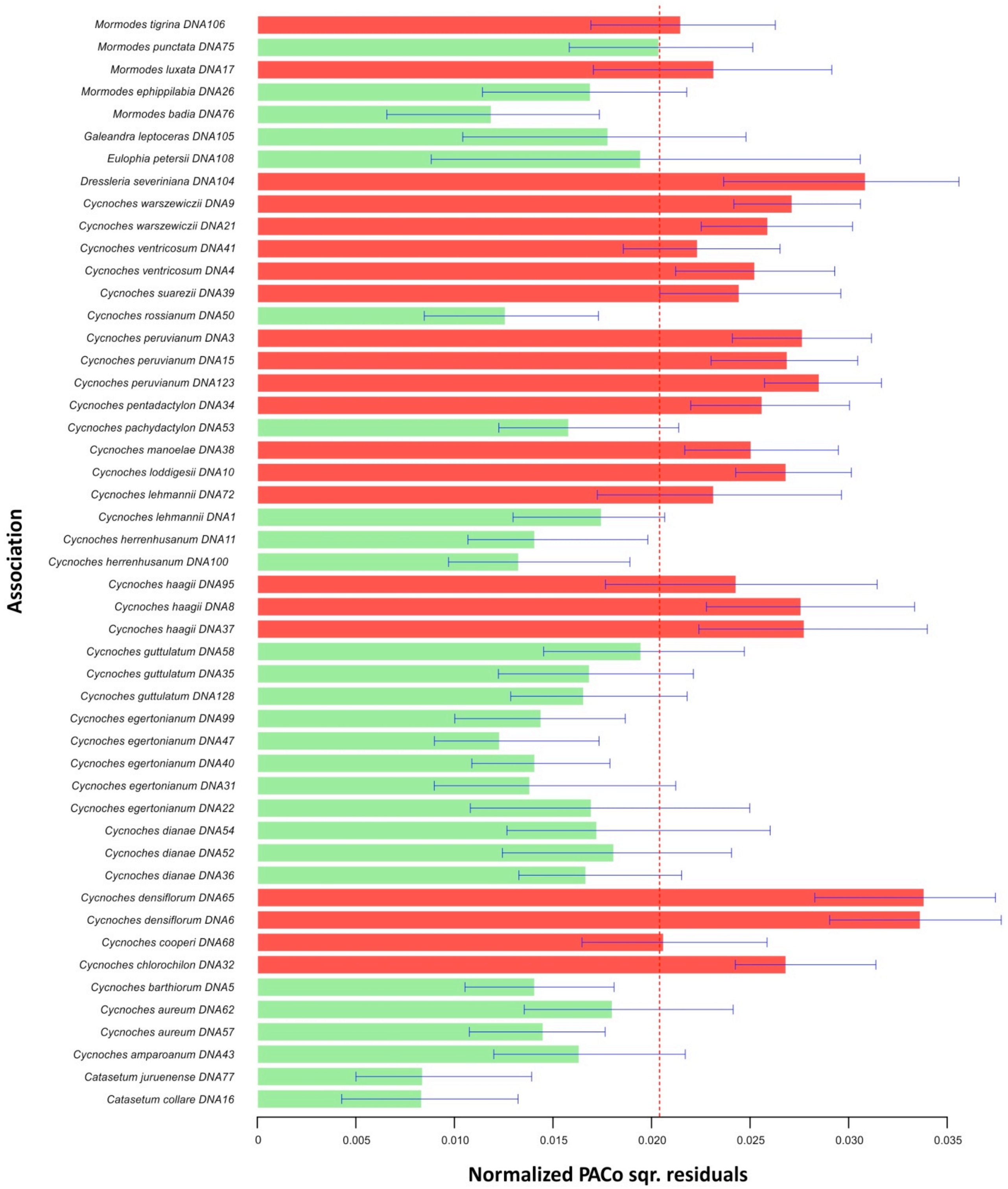

Figure S1.

Figure S2

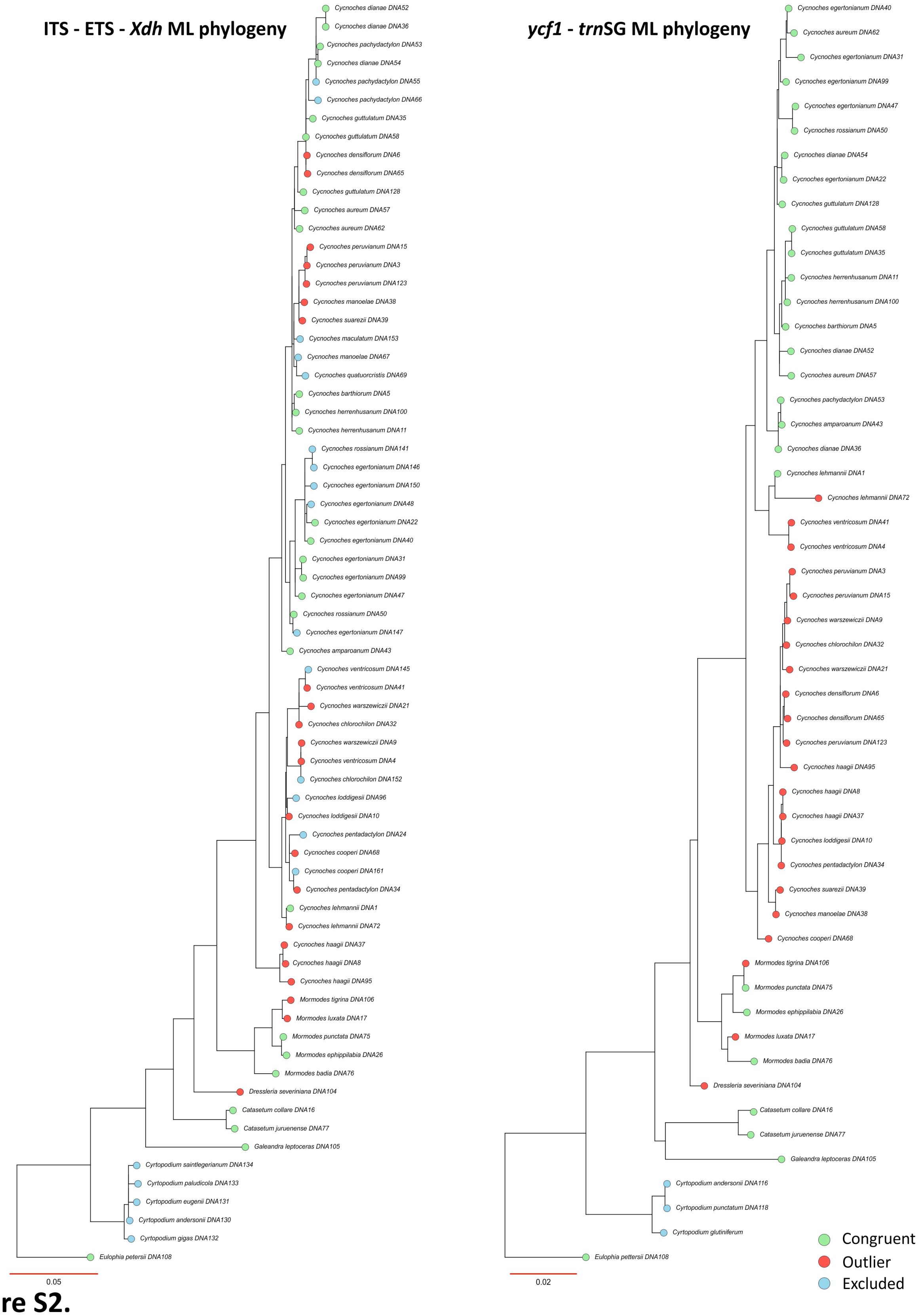

Figure S2.

Figure S3

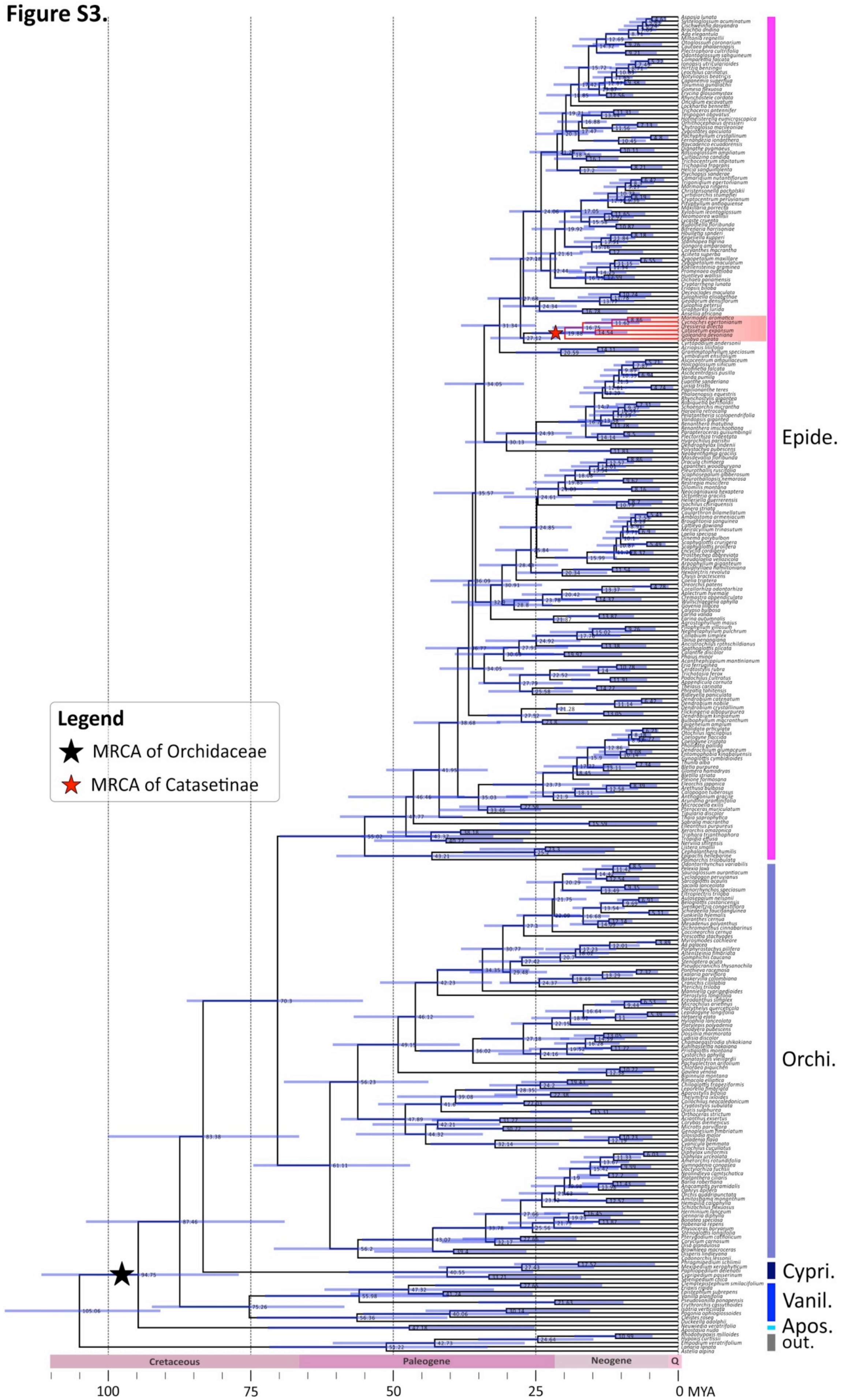
Figure S3.
Legend
MRCA of Orchidaceae
MRCA of Catasetinae
Epide.
Orchi.
Cypri.
Vanil.
Apos.
out.
Cretaceous
Paleogene
Neogene
Q
100
75
50
25
0 MYA

Figure S4

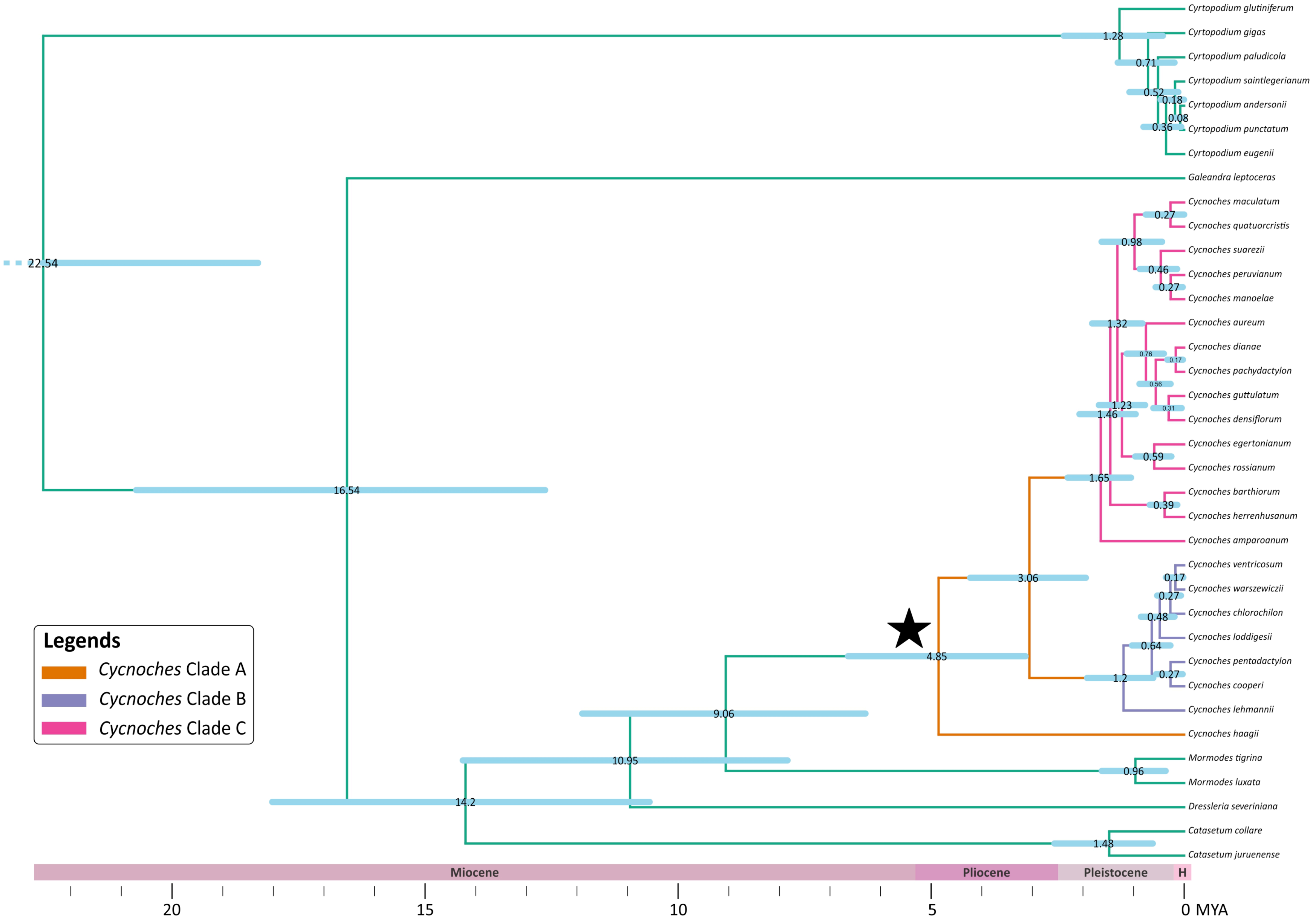

Figure S4.

Figure S5

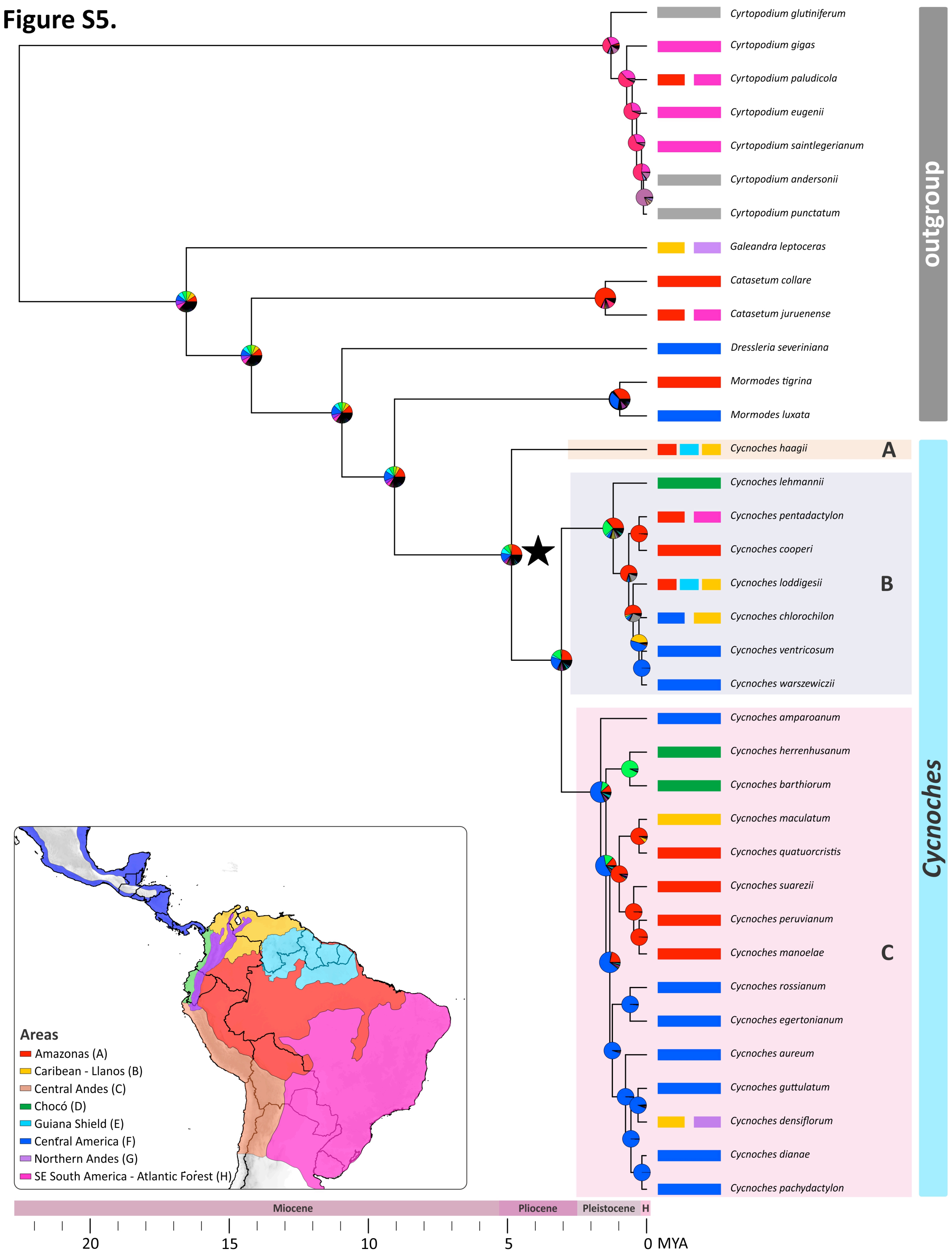
Figure S5.
Cyrtopodium glutiniferum
Cyrtopodium gigas
Cyrtopodium paludicola
Cyrtopodium eugenii
Cyrtopodium saintlegerianum
Cyrtopodium andersonii
Cyrtopodium punctatum
Galeandra leptoceras
Catasetum collare
Catasetum juruenense
Dressleria severiniana
Mormodes tigrina
Mormodes luxata
Cycnoches haagii
Cycnoches lehmannii
Cycnoches pentadactylon
Cycnoches cooperi
Cycnoches loddigesii
Cycnoches chlorochilon
Cycnoches ventricosum
Cycnoches warszewiczii
Cycnoches amparoanum
Cycnoches herrenhusanum
Cycnoches barthiorum
Cycnoches maculatum
Cycnoches quatuorcristis
Cycnoches suarezii
Cycnoches peruvianum
Cycnoches manoelae
Cycnoches rossianum
Cycnoches egertonianum
Cycnoches aureum
Cycnoches guttulatum
Cycnoches densiflorum
Cycnoches dianae
Cycnoches pachydactylon
A
B
C
outgroup
Cycnoches
Areas
Amazonas (A)
Caribean - Llanos (B)
Central Andes (C)
Chocó (D)
Guiana Shield (E)
Central America (F)
Northern Andes (G)
SE South America - Atlantic Forest (H)
Miocene
Pliocene
Pleistocene
H
20
15
10
5
0 MYA
